# Supplementary figures and images for: Cortical evoked activity is modulated by the sleep state in a ferret model of tinnitus. A cross-case study
Source: PLoS One. 2024 Dec 4;19(12):e0304306. doi: 10.1371/journal.pone.0304306 (PMC11616861; doi:10.1371/journal.pone.0304306)

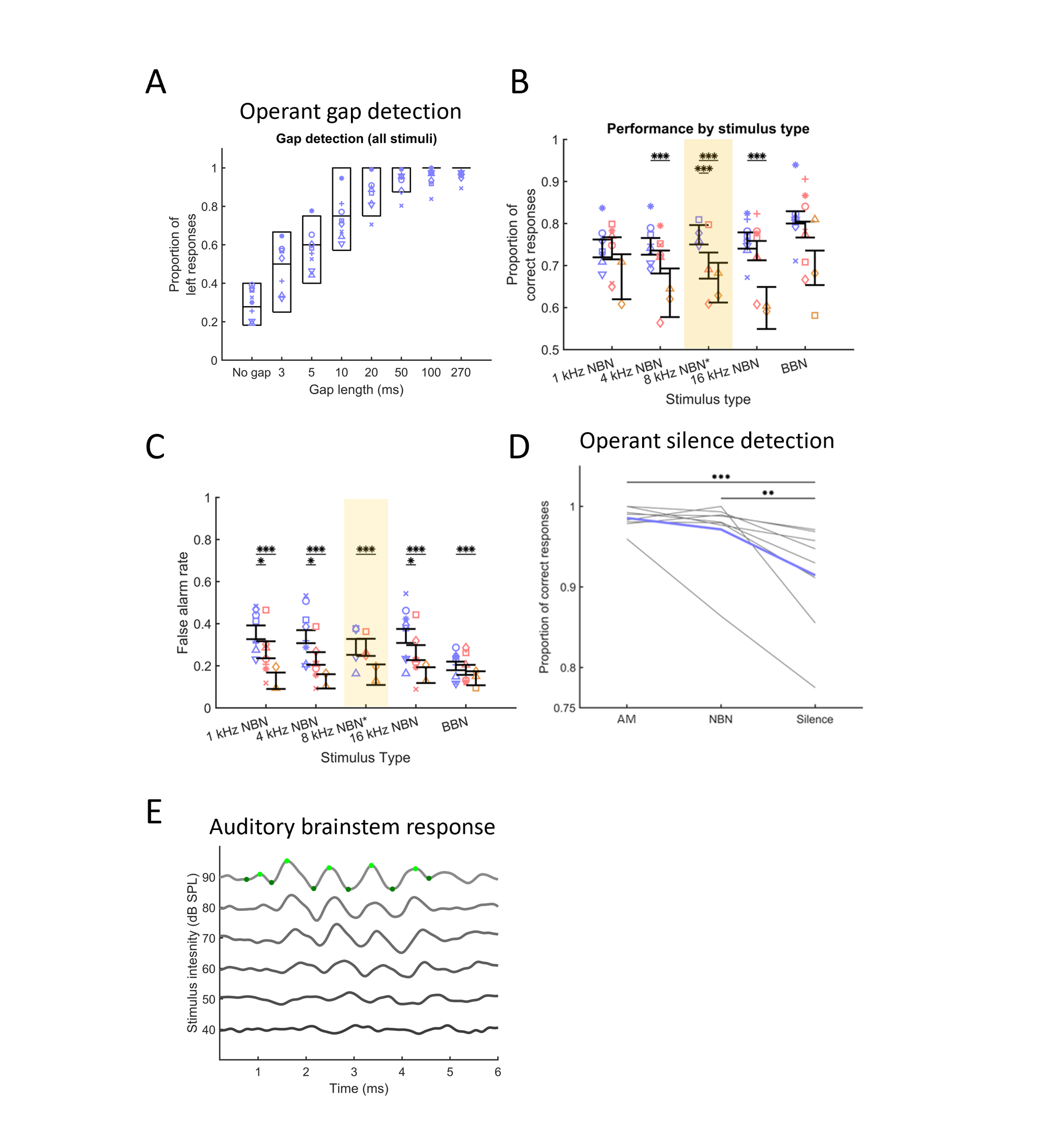

Supplement: S1 Fig — (A) Baseline performance in the operant gap detection task showing the proportion of left (-30°) responses by gap length (n = 7). Data encompass all stimuli (broadband noise, BBN, one octave narrow band noise, and one octave narrowband noise, NBN, centred at 1, 4, 8, 16 kHz). Left responses correspond to correct responses for gap stimuli and incorrect responses (false alarm) for no-gap stimuli. Symbols show individual animal means, horizontal lines median values, and boxes interquartile ranges across sessions. Triangle, square and circle symbols represent the animals in which electrophysiological recordings were made during sleep (ferrets 1–3 throughout the manuscript). (B) Operant gap detection, proportion of correct responses for each stimulus (1, 4, 8, 16 kHz NBN and BBN). Error bars represent the 95% confidence intervals. Timeline of testing in B and C is colour coded: blue, baseline, red one week after NOE and orange 6 months after NOE. (C) Operant gap detection, False alarm (FA) rate by stimulus type across time. FA = 1—proportion of correct responses in no gap trials. Markers represent the mean values from individual animals in panels A-C. (D) Baseline performance in the silence detection task. Proportion of correct responses in BBN, NBN, and silence trials. Grey lines show the mean values for each animal for each stimulus type, and the blue line depicts the mean across animals. Asterisks indicate statistical significance, ** p<0.01, *** p<0.001. (E) Exemplar trace of auditory brainstem response (ABR) to 8 kHz centred narrowband noise at baseline. Green markers depict peaks and throughs of ABR waves 1 to 4. (TIF) [file pone.0304306.s001.tif]

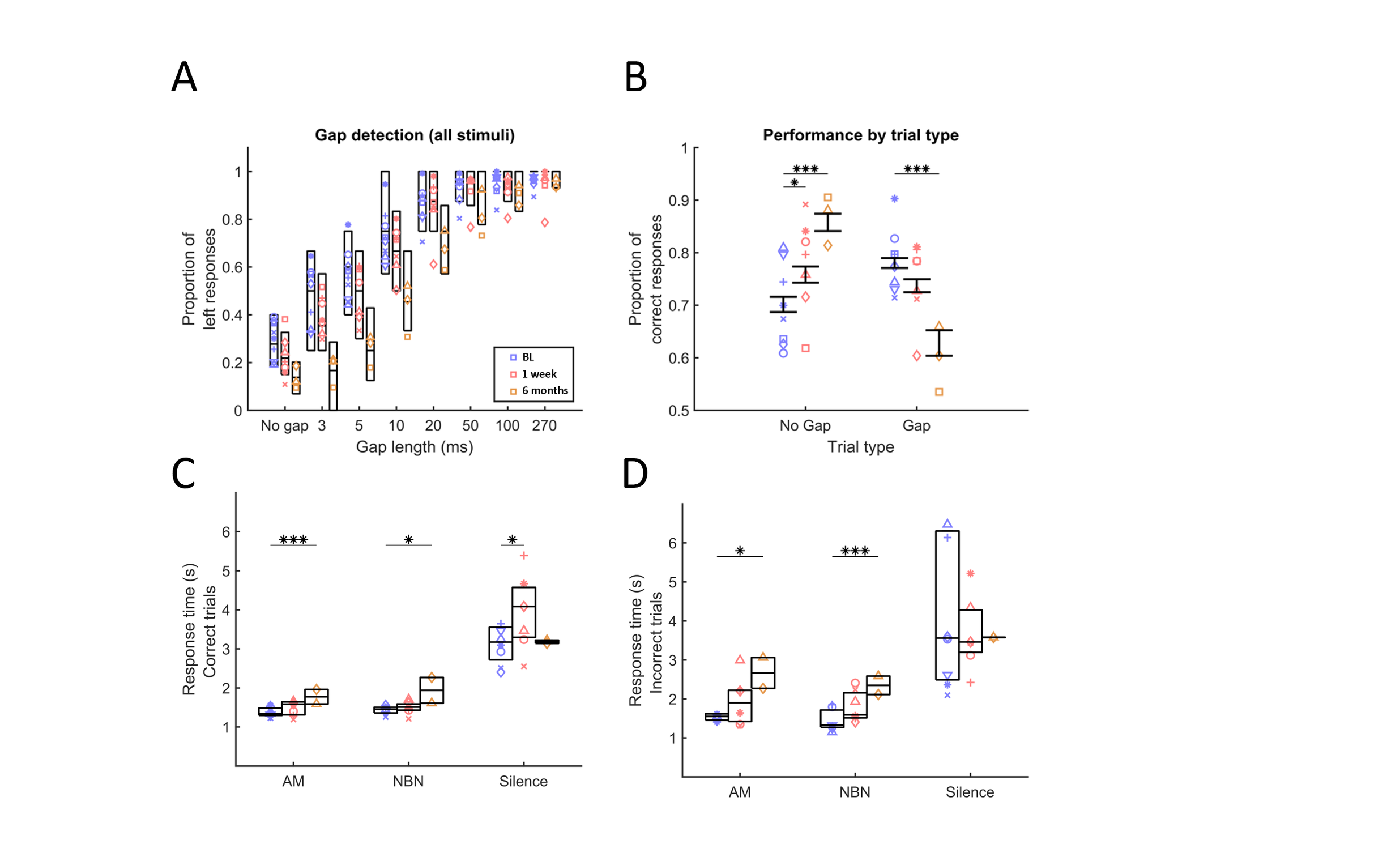

Supplement: S2 Fig — (A) Gap detection performance (left responses) by gap length across time (Baseline, and the two post-NOE assessments, One week and Six months). Left responses are correct responses in gap trials and incorrect responses in no-gap trials. (B) Proportion of correct responses by trial type (no gap and gap) across time, colour coded. (C, D) Response times in the Silence detection paradigm. Response times in NBN, BBN and silence trials for correct responses (C) and for incorrect responses (D). Asterisks represent statistical significance *p<0.05, **p<0.01, ***p<0.001. Triangle, square and circle symbols represent the animals in which electrophysiological recordings were made during sleep. (TIF) [file pone.0304306.s002.tif]

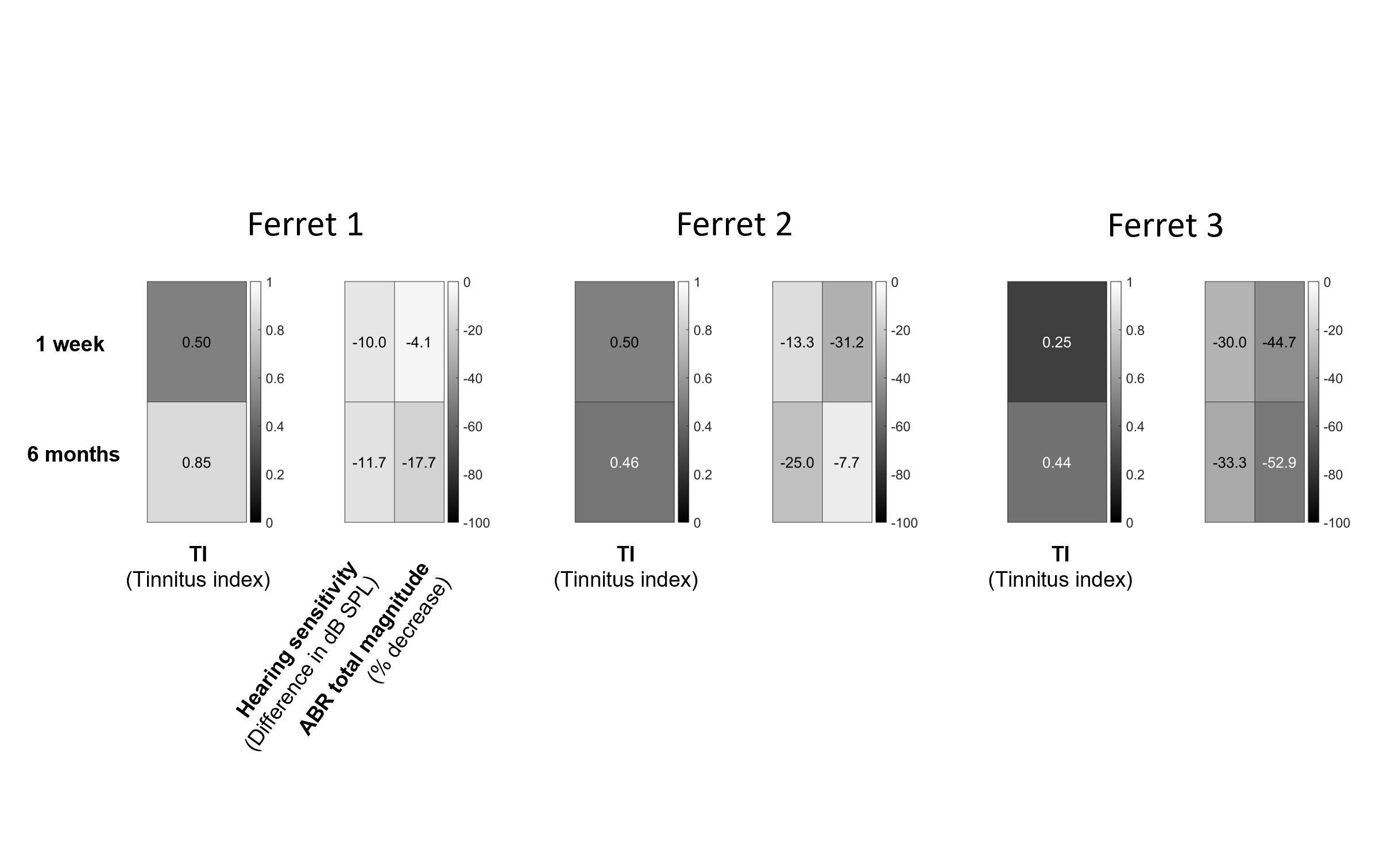

Supplement: S3 Fig — Tinnitus index (TI) and hearing loss relative to baseline (BL) (see Methods). Each panel depicts one ferret. Panels left to right: Ferret 1, Ferret 2, Ferret 3. The tinnitus index (TI) for each animal was based on behavioural impairments in gap and silence detection. Hearing loss was defined as changes in hearing sensitivity (based on ABR thresholds) and changes in ABR magnitude. The numbers and grey colour coding correspond to the size of change relative to BL of the measures at one week and 6 months after noise overexposure. (TIF) [file pone.0304306.s003.tif]

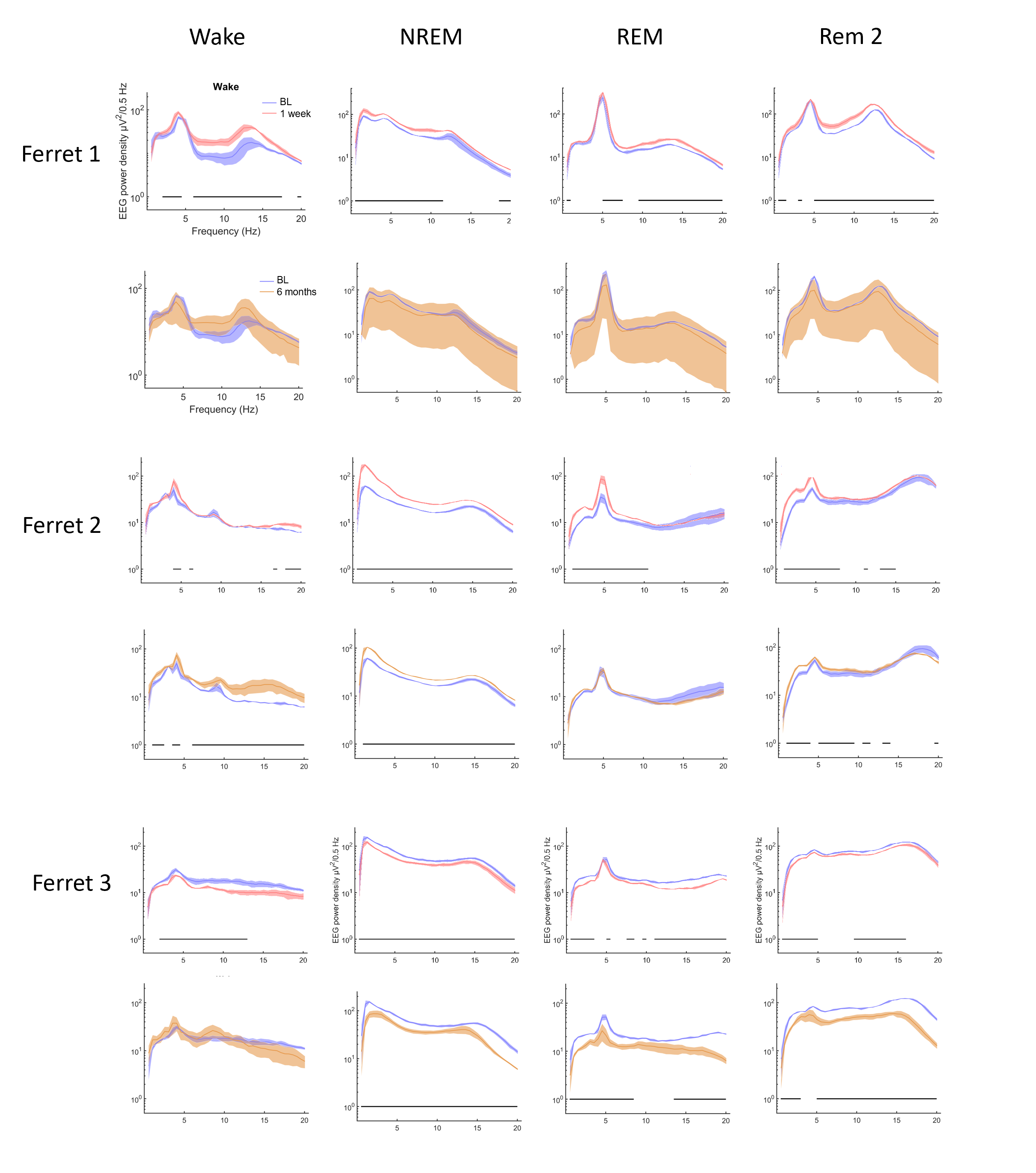

Supplement: S4 Fig — For each ferret, the plots on the upper row show the baseline (BL) and One week EEG spectra, while on the lower row show the BL and Six months EEG spectra for the frontal EEG derivation. Spectra are averages ± standard errors of the mean based on recordings over 48 hours per condition and displayed in 0.5 Hz bins. Originally, average 24 h EEG spectra were produced with a resolution of 0.25 Hz (See Fig 2E). For comparison between conditions, data in each 0.25 Hz bin were merged into 0.5 Hz bins, producing 2 datapoints per 0.5 Hz bin per 24 h recording, and ultimately in 4 datapoints per 0.5Hz bin since EEG spectra obtained during both 24 h periods within the same condition were combined. Two-way ANOVA (factors condition and frequency bin), Tukey’s multiple comparisons; horizontal solid black lines over the x axis indicate statistical significance p < 0.05. (TIF) [file pone.0304306.s004.tif]

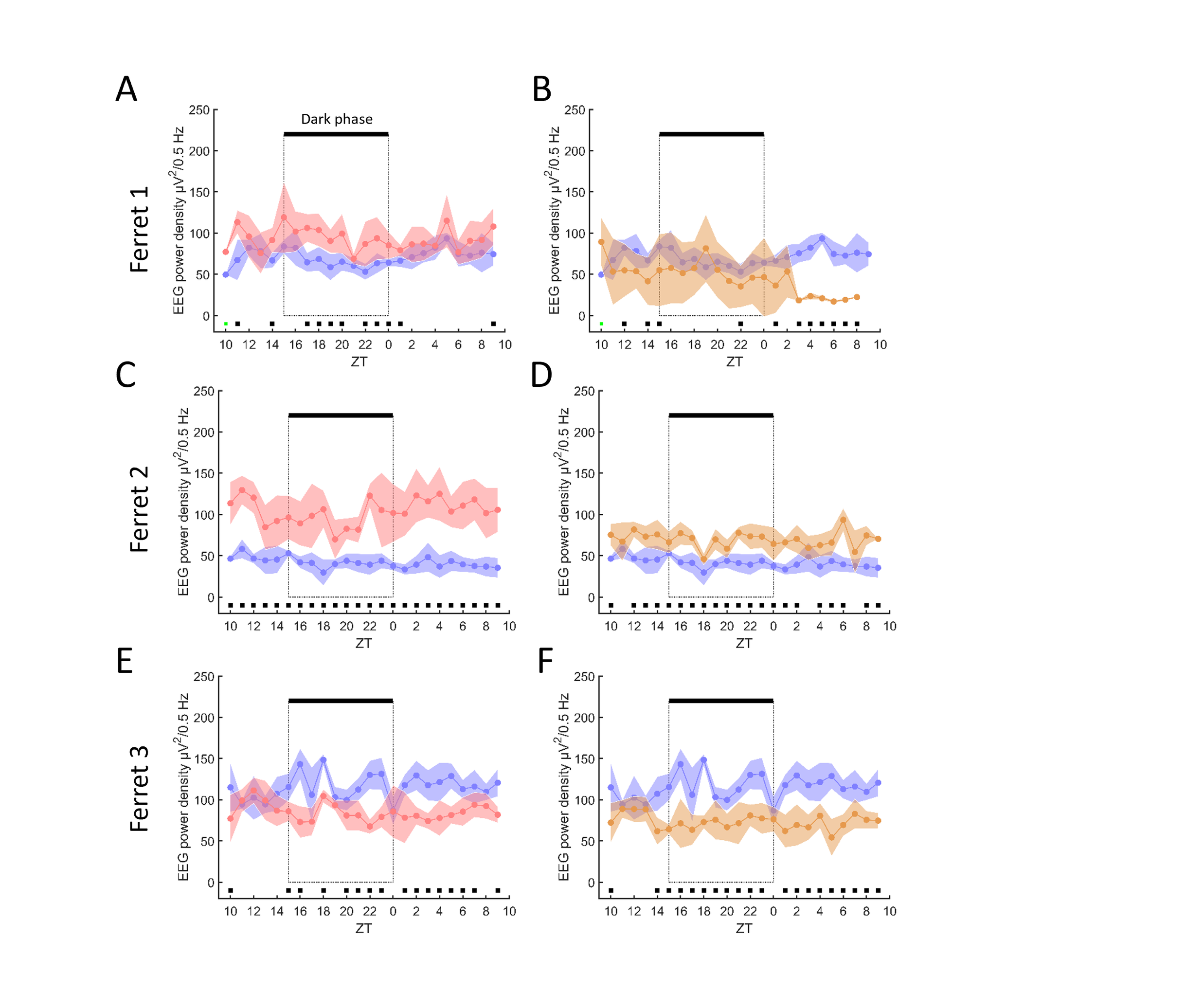

Supplement: S5 Fig — (BL, blue, One week, red, Six months, orange). NREM slow wave activity (SWA) was calculated per 15-min interval, before being merged into 1-hour intervals, resulting in 4 datapoints per 1-hour bin per 24 hours and 8 datapoints per condition (consisting of two 24h recording periods). Shaded error bars are standard deviations. Two-way ANOVA (factors condition and time interval), Tukey’s multiple comparisons; black square markers indicate differences at p < 0.05. Green squares in panels A and B mark time intervals with less than 3 data points in either condition. Times are expressed in zeitgeber time (ZT), time 0 corresponds to the start of the light period. (TIF) [file pone.0304306.s005.tif]

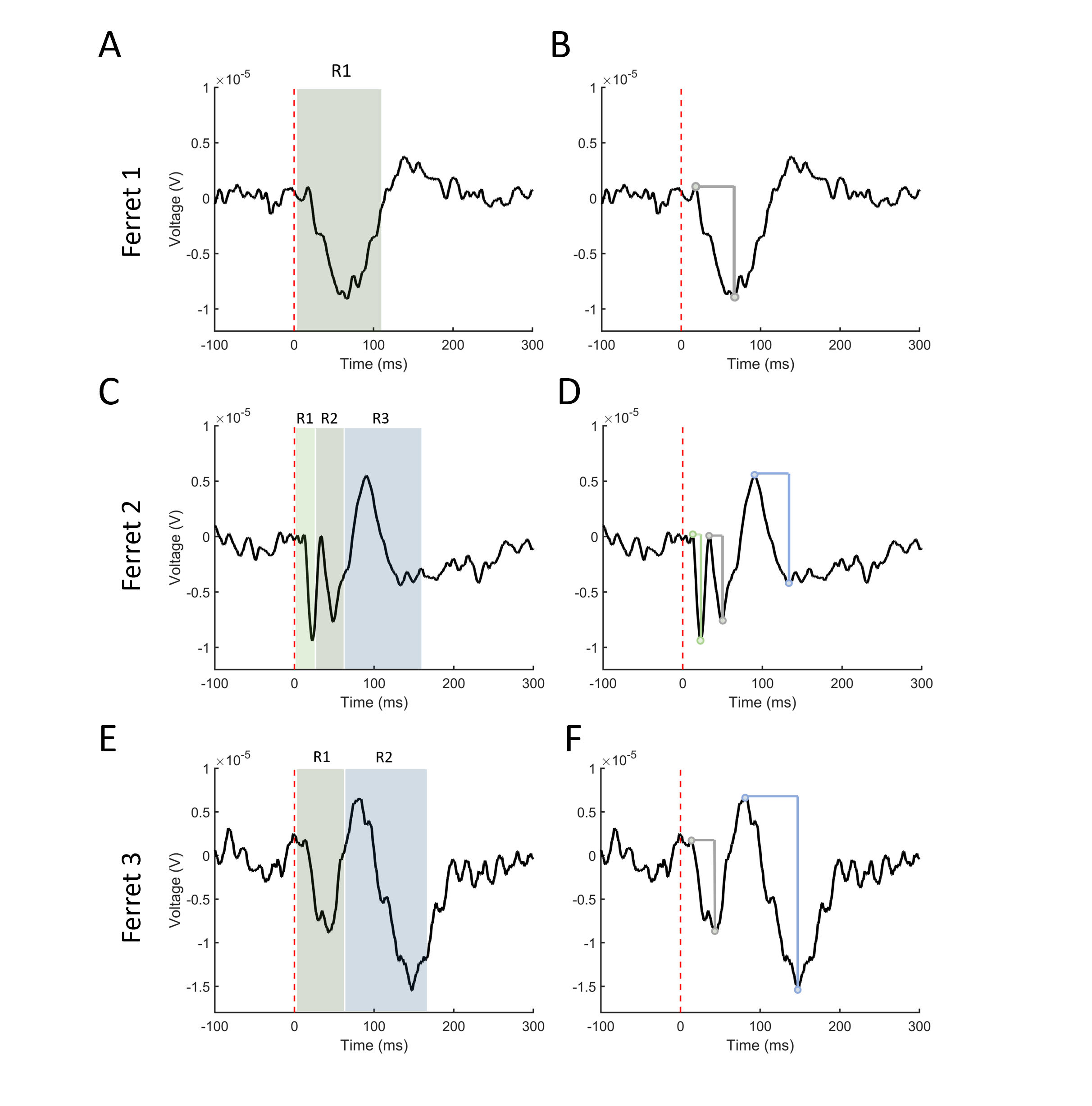

Supplement: S6 Fig — Each row depicts one ferret (ferrets 1–3). Left column (A,C,E) time windows for response components. Average occipital EEG evoked potential during BL recordings with an outline of the time windows where the response components (R1, R2, R3) were defined. Similar windows were applied for the frontal EEG signal (not shown). Note that a different number of response windows was defined for different animals. Right column (B,D,F) Definition of response magnitudes. A custom MATLAB algorithm selected the maximum value within each response window and the subsequent minimum to compute the response magnitude for each response component. (TIF) [file pone.0304306.s006.tif]

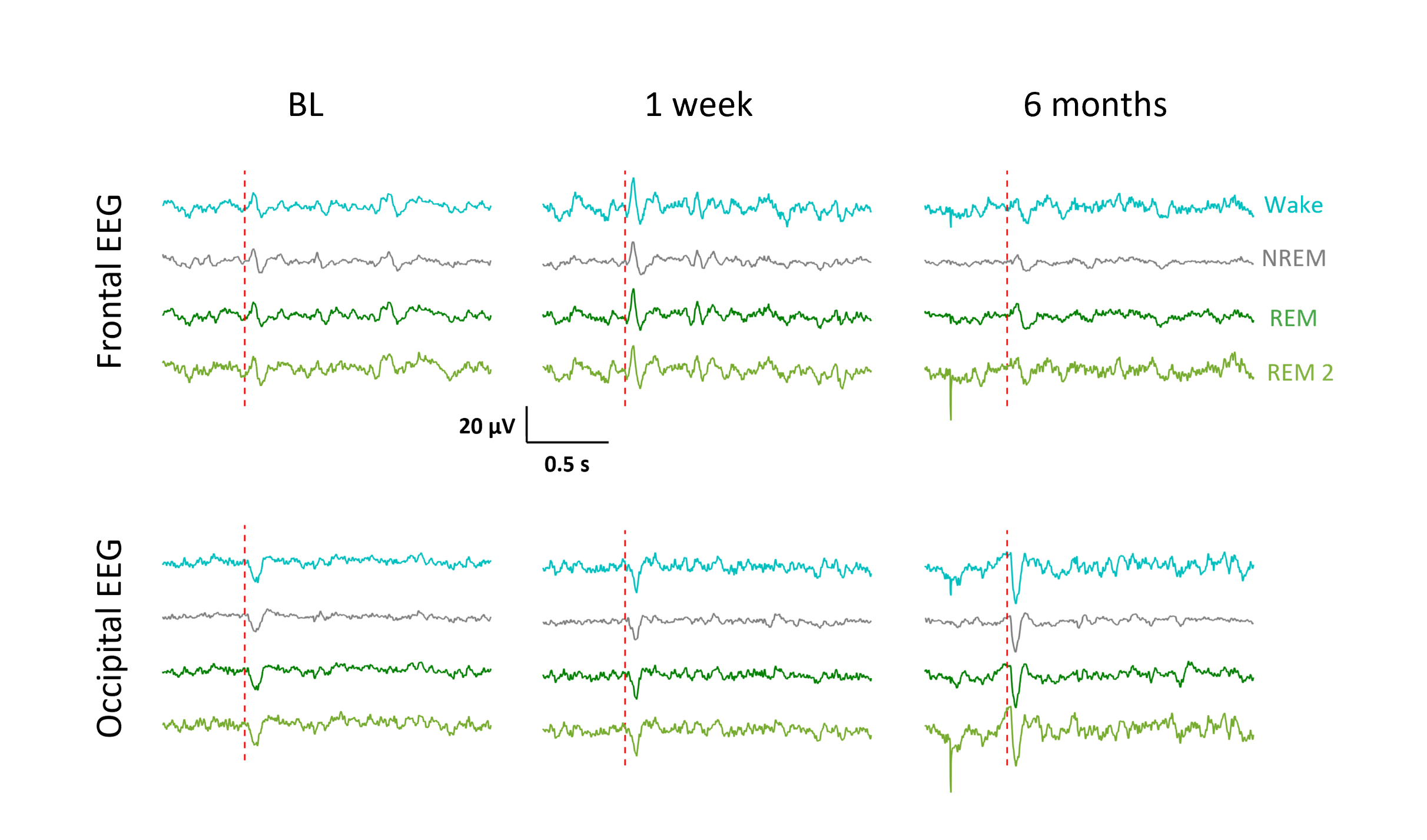

Supplement: S7 Fig — Auditory evoked responses for EEG frontal (top panels) and EEG occipital derivations (bottom panels) during the BL (baseline), 1 week and 6 months conditions. The dotted red vertical line in each panel depicts the stimulus onset. The averages displayed in this figure are based on snippets of raw recording data after the exclusion of signal artefacts (see Methods: vigilance state scoring) and are averages over signals for all stimulus types (820 ms narrow-band sounds centred around 1, 4, 8 and 16 kHz with a 38 ms silent gap at the centre of the stimulus,) and multiple levels (40, 50, 60 and 65 dB SPL). (TIF) [file pone.0304306.s007.tif]

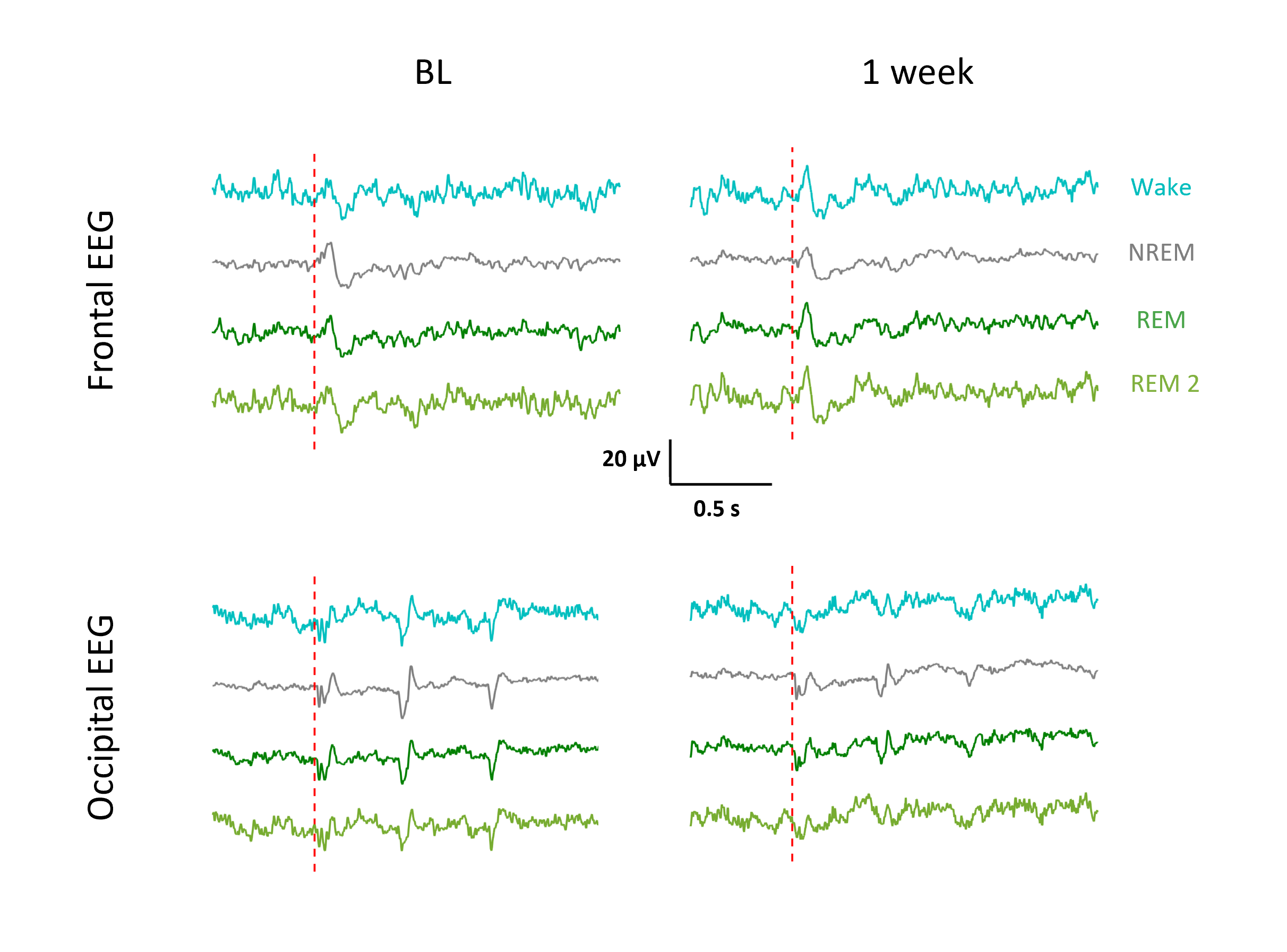

Supplement: S8 Fig — Auditory evoked responses for EEG frontal (top panels) and EEG occipital derivations (bottom panels) during the BL (base line) and 1 week condition. The signal quality in 6 months condition was insufficient for quantitative analysis. The dashed red vertical line in each panel depicts the stimulus onset. The averages displayed in this figure are based on snippets of raw recording data after the exclusion of signal artefacts (see Methods: vigilance state scoring) and are averages over signals for all stimulus types (820 ms narrow-band sounds centred around 1, 4, 8 and 16 kHz with a 38 ms silent gap at the centre of the stimulus,) and multiple levels (40, 50, 60 and 65 dB SPL). (TIF) [file pone.0304306.s008.tif]

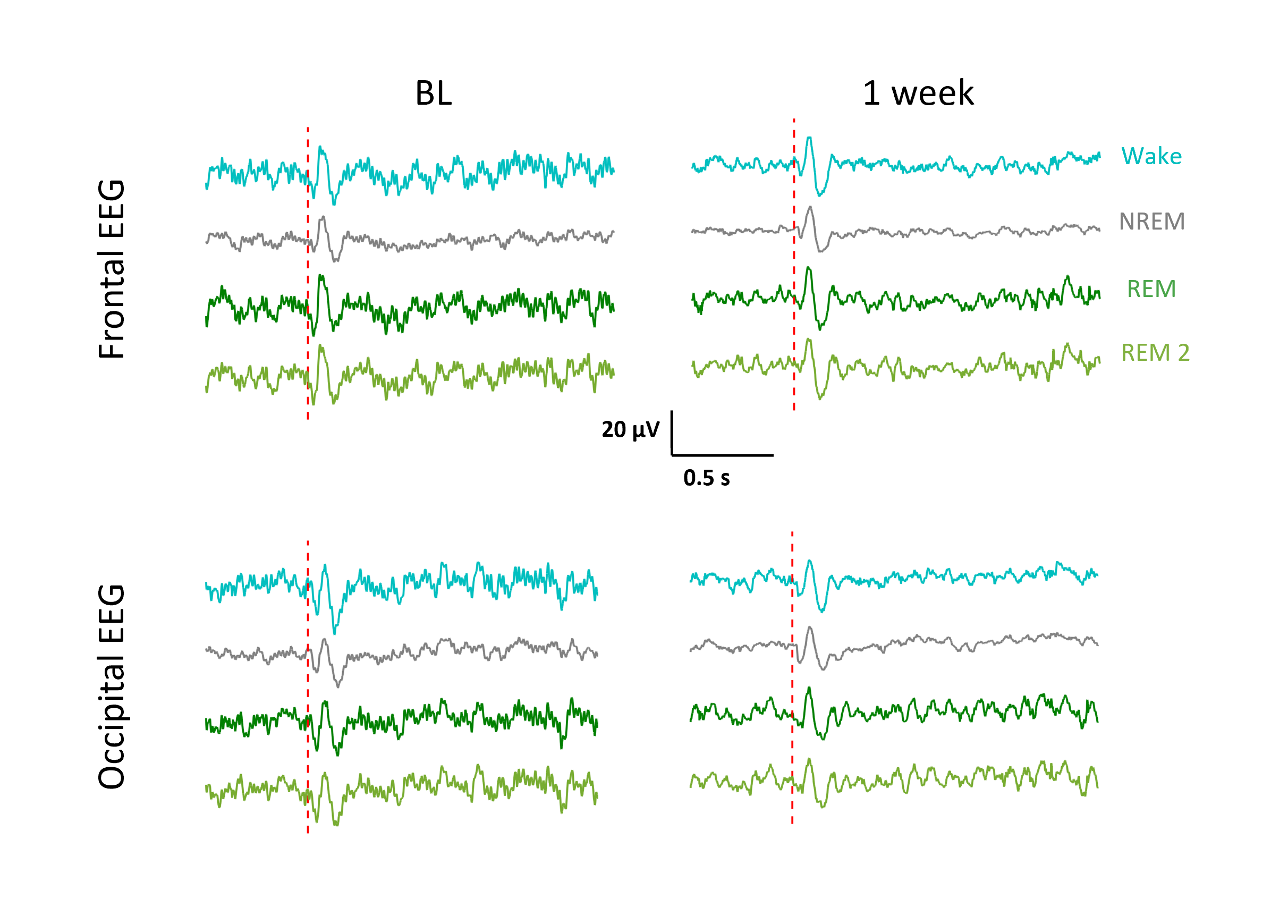

Supplement: S9 Fig — Auditory evoked responses for EEG frontal (top panels) and EEG occipital derivations (bottom panels) during the BL (baseline) and one week condition. The signal quality in six months condition was insufficient for quantitative analysis. The dashed red vertical line in each panel depicts the stimulus onset. The averages displayed in this figure are based on snippets of raw recording data after the exclusion of signal artefacts (see Methods: vigilance state scoring) and are averages over signals for all stimulus types (820 ms narrow-band sounds centred around 1, 4, 8 and 16 kHz with a 38 ms silent gap at the centre of the stimulus,) and multiple levels (40, 50, 60 and 65 dB SPL). (TIF) [file pone.0304306.s009.tif]

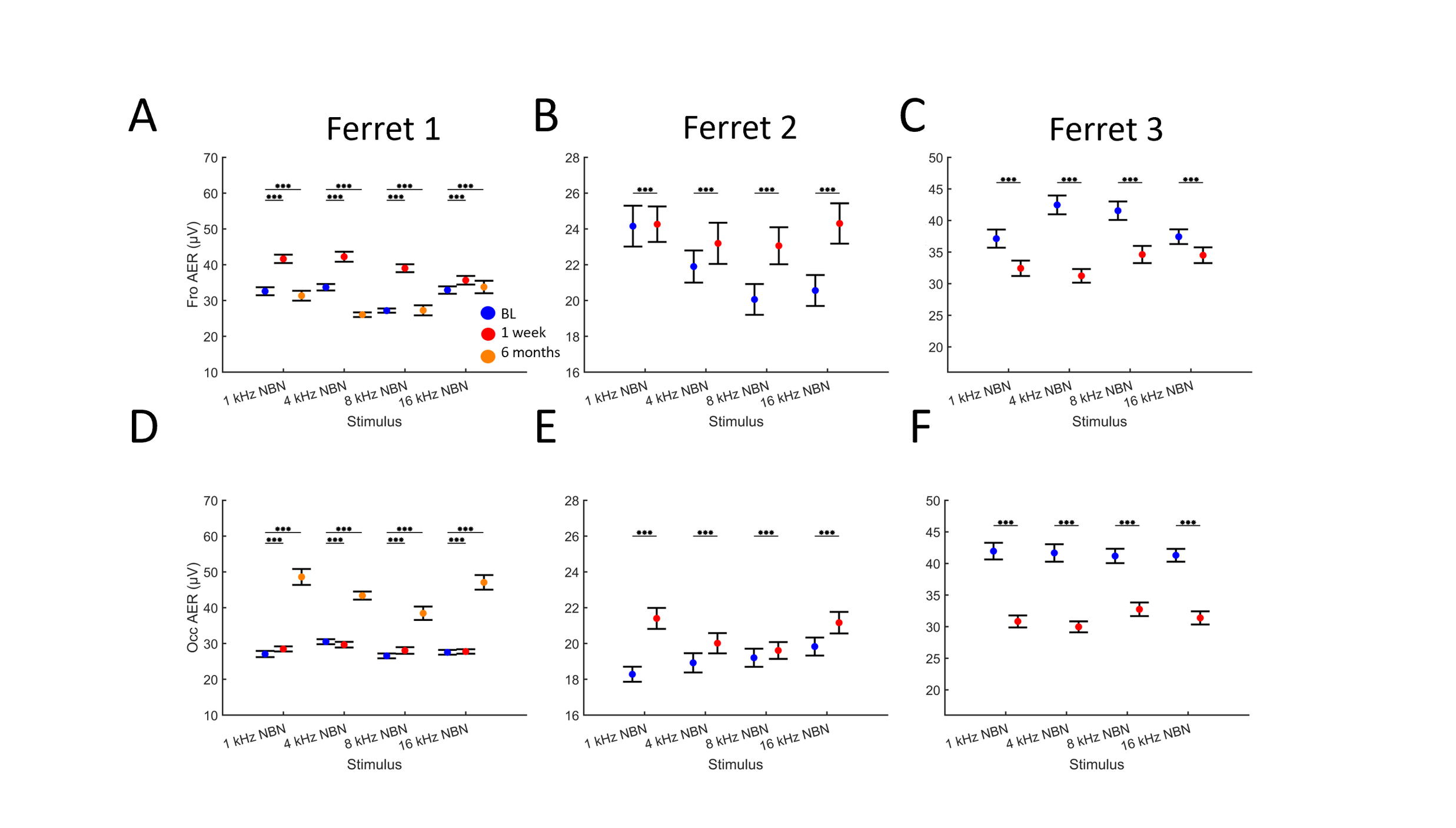

Supplement: S10 Fig — Averages across all vigilance states for ferrets 1–3 organised in columns and for the different EEG configurations, (A-C) Frontal EEG evoked response (AER) for all stimuli before (baseline, blue) and after NOE (1 week, red; 6 months, orange) and (D-F) for occipital EEG. Data are bootstrapped averages across response components, vigilance state and sound level (see Methods for details). Error bars are standard errors. All group comparisons differed at p<0.001 (***, GLMM, factor ‘condition’). AER magnitude for each stimulus. Averages across all vigilance states. Each panel depicts one ferret. Panels left to right: Ferret 1, Ferret 2, Ferret 3. All group comparisons differed at p<0.001 (GLMM, factor ‘condition’). (TIF) [file pone.0304306.s010.tif]

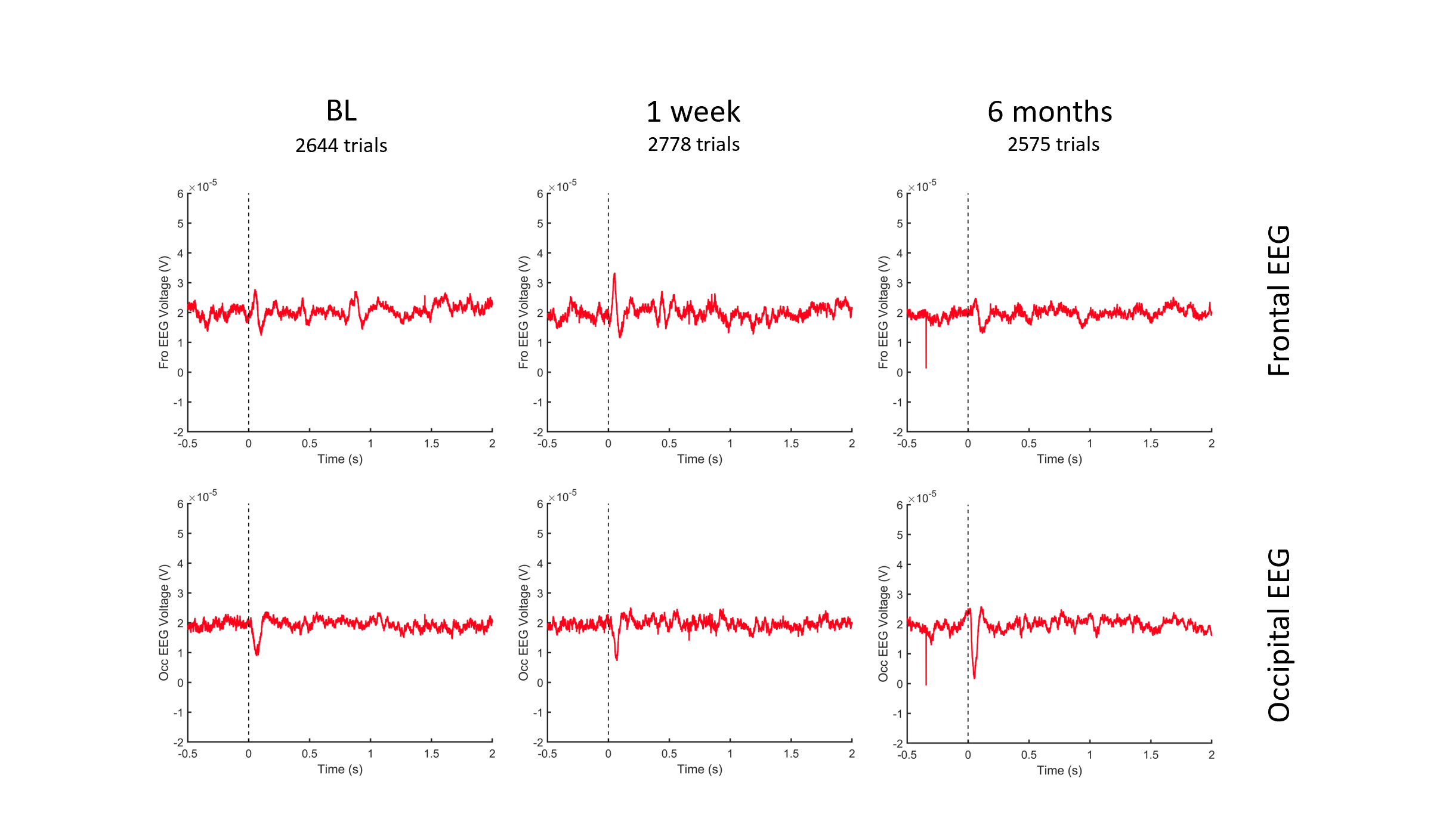

Supplement: S11 Fig — Number of trials contributing to the average are indicated above each panel. Note that trials falling into epochs scored as ‘artefact’ during the manual scoring procedure were not included. (TIF) [file pone.0304306.s011.tif]

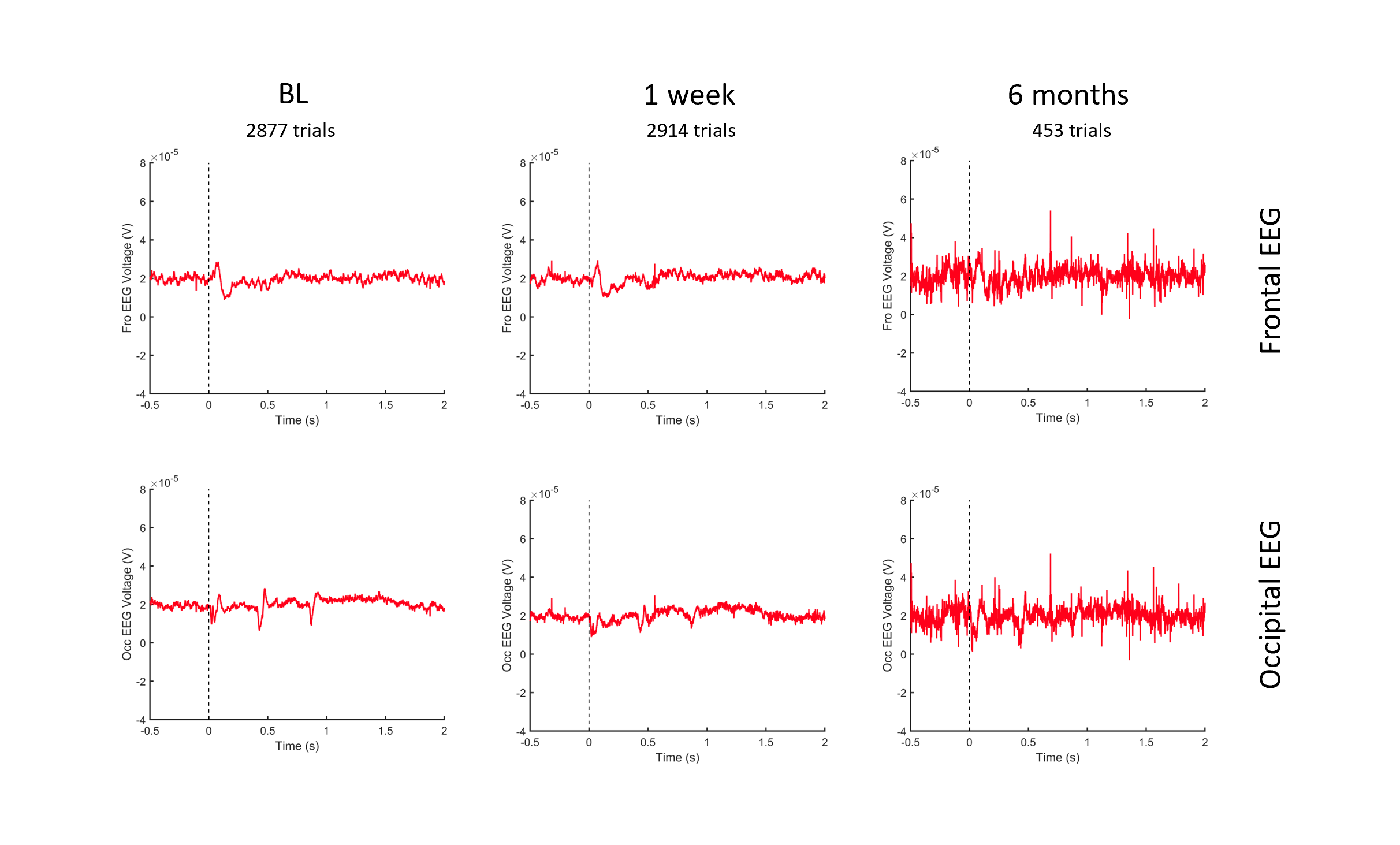

Supplement: S12 Fig — Number of trials contributing to the average are indicated above each panel. Note that trials falling into epochs scored as ‘artefact’ during the manual scoring procedure were not included. To produce this figure and because signals in the six months condition were generally of lower quality, only trials where the signal’s standard deviation did not exceed double the average standard deviation were included. (TIF) [file pone.0304306.s012.tif]

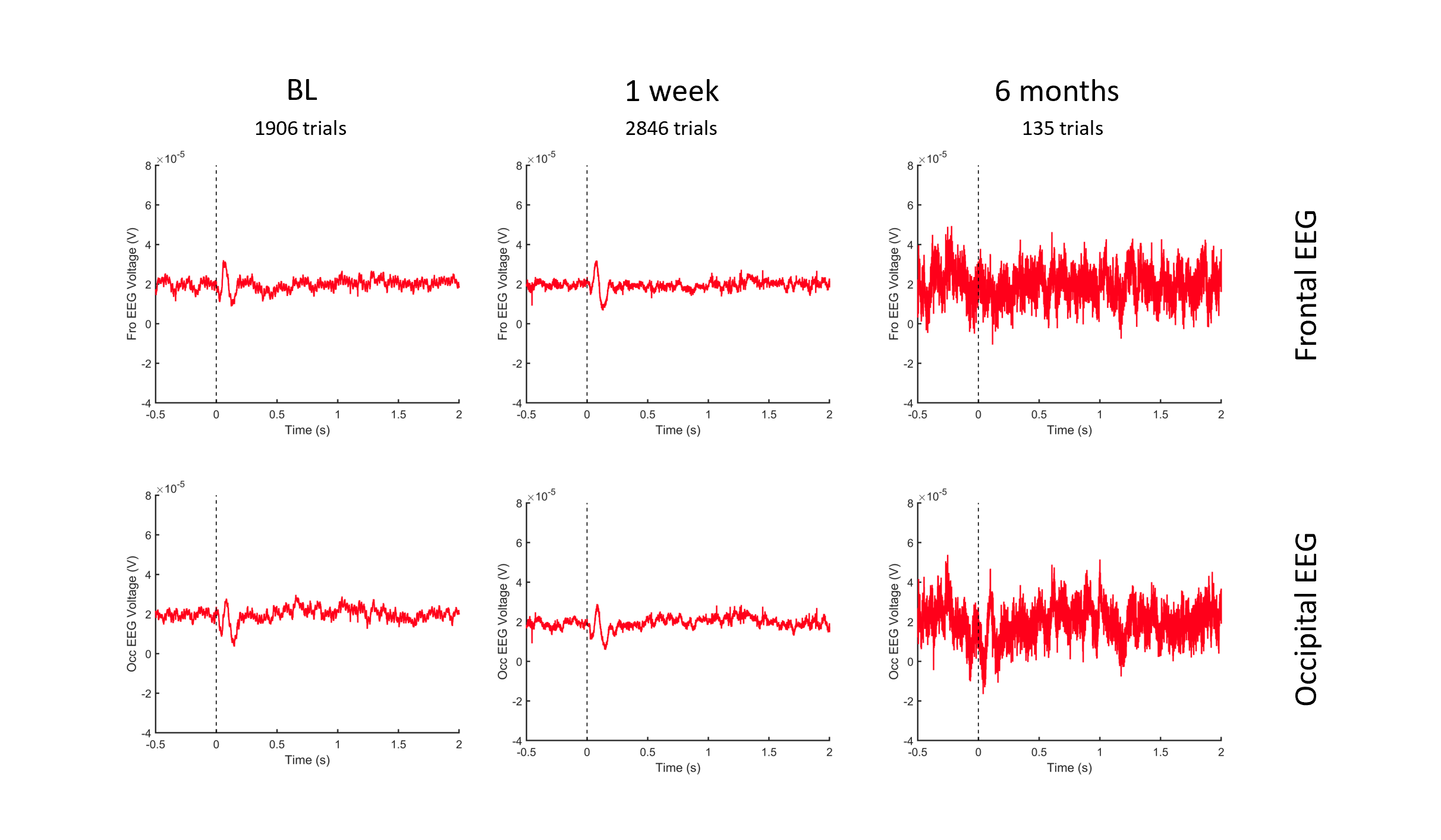

Supplement: S13 Fig — Number of trials contributing to the average are indicated above each panel. Note that trials falling into epochs scored as ‘artefact’ during the manual scoring procedure were not included. To produce this figure and because signals in the Six months condition were generally of lower quality, only trials where the signal’s standard deviation did not exceed double the average standard deviation were included. (TIF) [file pone.0304306.s013.tif]

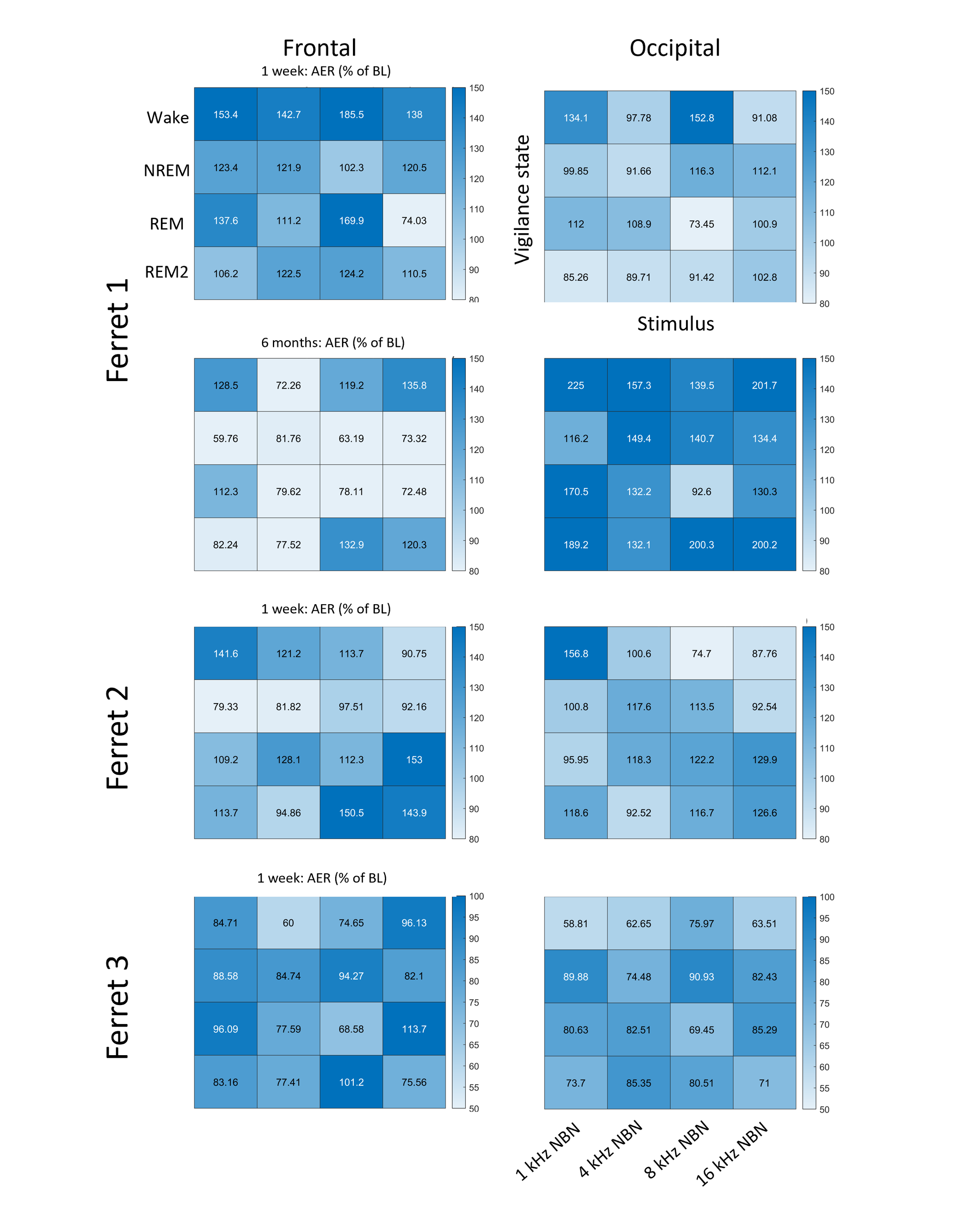

Supplement: S14 Fig — Values are average auditory evoked response magnitudes as a percentage of the baseline condition for the frontal EEG signal (left panels) and for the occipital EEG signal (right panels). In each panel, data are averaged across stimulus levels and response windows. The x-axis represents stimuli, the y-axis represents the vigilance states (Wake, NREM, REM, REM2). The first two rows in the figure (consisting of 4 panels) correspond to Ferret 1, the third row to Ferret 2, and the last row to Ferret 3. For all animals average auditory evoked response magnitudes for 1 week were calculated. In addition, magnitudes for 6 months were only calculated for Ferret 1 and are shown in the second row of figure panels. (TIF) [file pone.0304306.s014.tif]

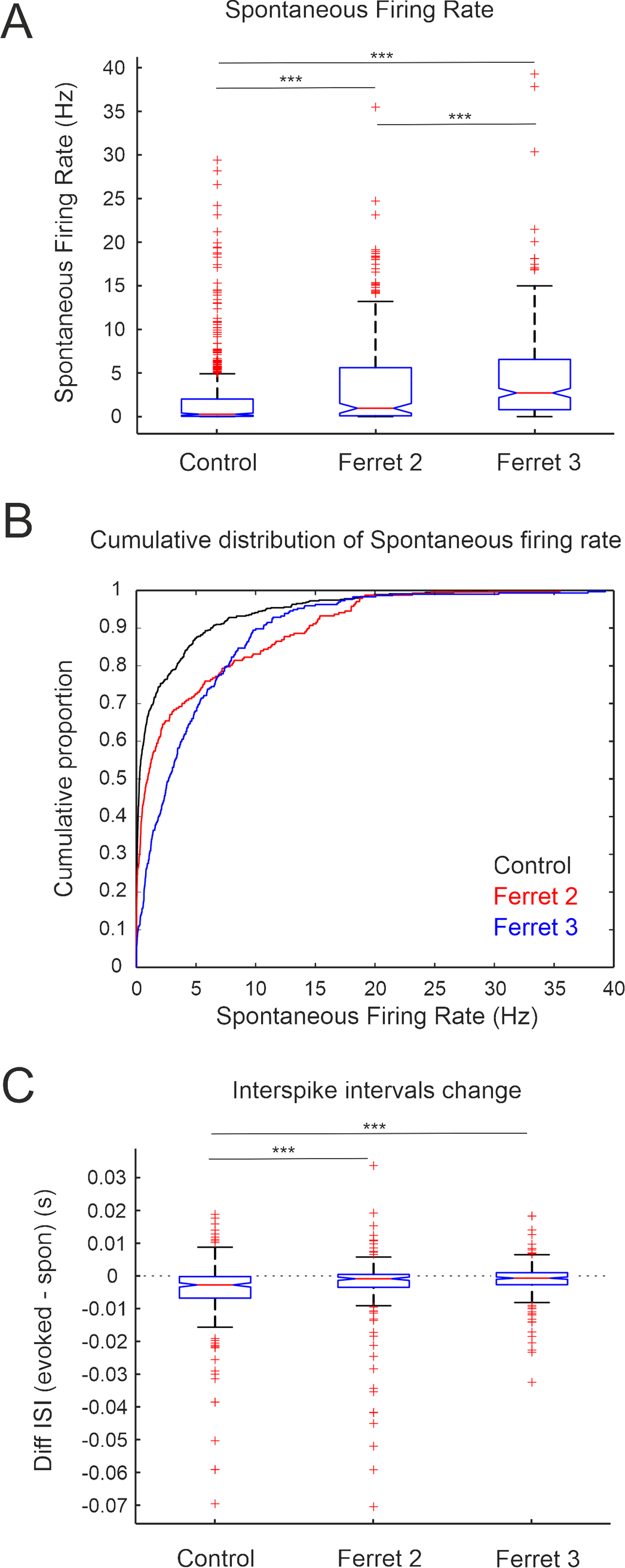

Supplement: S15 Fig — (A) Spontaneous activity of acoustically-responsive neurons for a Control ferret (n = 543 units) and two NOE animals Ferret 2 (n = 237) and Ferret 3 (n = 294). NOE animals exhibited significantly higher spontaneous activity (Kruskal-Wallis, χ2 (2,1073) = 122.83; p<0.0001) and a greater variability as indicated by the larger interquartile range. In each box, the central mark indicates the median, and the bottom and top edges of the box indicate the 25th and 75th percentiles, respectively. The whiskers extend to the most extreme data points not considered to be outliers, and the outliers are plotted individually using the ’+’ marker symbol. (B) Cumulative distribution of cortical units according to their spontaneous firing rates for the three ferrets. Cumulative functions for NOE ferrets showed a broader distribution skewed towards higher rates. (C) Difference between evoked (to broadband noise presentation) and spontaneous interspikes intervals (ISI). Although a reduction of the ISI with stimulus presentation was expected because of increased activity following the stimulus, this reduction was significantly more marked in the control ferret than after NOE (Kruskal-Wallis, χ2 (2,629) = 28.97; p<0.0001), suggesting that the temporal properties of the responses was less precise in NOE ferretsª. (TIF) [file pone.0304306.s015.tif]
