# Supplementary material for: Cortical evoked activity is modulated by the sleep state in a ferret model of tinnitus. A cross-case study
Source: PLoS One. 2024 Dec 4;19(12):e0304306. doi: 10.1371/journal.pone.0304306 (PMC11616861; doi:10.1371/journal.pone.0304306)
Supplement: Attachment — Submitted filename: Response to the reviewers 270724.docx [file pone.0304306.s016.docx]

[Note: HTML markup is below. Please do not edit.]

Reviewers' comments:

Reviewer's Responses to Questions

**Comments to the Author**

1. Is the manuscript technically sound, and do the data support the conclusions?

The manuscript must describe a technically sound piece of scientific research with data that supports the conclusions. Experiments must have been conducted rigorously, with appropriate controls, replication, and sample sizes. The conclusions must be drawn appropriately based on the data presented.

Reviewer #1: Partly

Reviewer #2: Partly

2. Has the statistical analysis been performed appropriately and rigorously?

Reviewer #1: Yes

Reviewer #2: No

3. Have the authors made all data underlying the findings in their manuscript fully available?

The [PLOS Data policy](http://www.plosone.org/static/policies.action#sharing) requires authors to make all data underlying the findings described in their manuscript fully available without restriction, with rare exception (please refer to the Data Availability Statement in the manuscript PDF file). The data should be provided as part of the manuscript or its supporting information, or deposited to a public repository. For example, in addition to summary statistics, the data points behind means, medians and variance measures should be available. If there are restrictions on publicly sharing data—e.g. participant privacy or use of data from a third party—those must be specified.

Reviewer #1: Yes

Reviewer #2: No

4. Is the manuscript presented in an intelligible fashion and written in standard English?

PLOS ONE does not copyedit accepted manuscripts, so the language in submitted articles must be clear, correct, and unambiguous. Any typographical or grammatical errors should be corrected at revision, so please note any specific errors here.

Reviewer #1: Yes

Reviewer #2: Yes

5. Review Comments to the Author

Please use the space provided to explain your answers to the questions above. You may also include additional comments for the author, including concerns about dual publication, research ethics, or publication ethics. (Please upload your review as an attachment if it exceeds 20,000 characters)

**Reviewer #1**: In this study, Milinski et al establish a behavioral model of noise-overexposure induced tinnitus in the adult female ferret, as verified by operant gap detection and silence detection paradigms. They combine their behavioral testing with chronic electrophysiological recordings to measure vigilance states before and after noise overexposure and relate the results to the behavioral evidence of tinnitus and hearing impairment. The premise of the study, which is that (1) the ferret, given its lower frequency hearing range compared to mice, long lifespan and ability to perform complex behavior tasks, is a valuable model for human subjective tinnitus, and (2) the ferret is also amenable to chronic electrophysiological and behavioral testing of sleep/wake states, is an appropriate model to investigate the oft-reported connection between tinnitus and sleep disturbance in humans, is sound. The topic is of broad interest to clinicians and basic researchers studying both sleep and sensory disorders, and the mechanistic connections between hearing impairment and sleep are understudied despite emerging attention focused on sound-induced recruitment of arousal systems in wakefulness. Therefore, the study is timely and interesting.

R1#1. We are grateful for the reviewer’s positive comments.

The authors highlight the bi-directional interaction between tinnitus and sleep, which while intriguing, is not strongly supported by the evidence. A major reason for this is the variability in the admittedly small group of animals tested. There is certainly value in testing a smaller cohort of animals over long time periods, but part of this strength is leveraged by testing multiple times at baseline prior to NOE. A major weakness of the study is the inability of the reader to understand the variability expected in sleep architecture, tinnitus behavior, and EAPs at baseline pre-exposure, in addition to the variability that could be expected over a 6 month testing period in a sham-exposed animal. A sham group would experience similar levels of signal degradation from an electrode, behavioral variability, and stress due to surgical or exposure procedures, but presumably without tinnitus.

R1#2. We understand the reviewer concerns and we have now provided an analysis of baseline variability in sleep architecture (see next comment R1#3).

We would like to add that although ferrets present individual variability in their behaviour, the behaviour does not usually degrade over time. Our Home Office animal licence allow us to run behavioural testing in adult ferrets for 30 months, which starts when they are young adults, about 1 year old. We have shown that behavioural performance changes little over this long period and is not affected by long, even consecutive surgeries. For example, in Bajo et al. (2010) we used two consecutive cranial surgeries under general anaesthesia to selectively ablate auditory corticocollicular neurons, and showed with specific controls having the same surgery but in different brain areas, that the surgery did not produce any deficits in sound localization accuracy (Bajo et al., Nat Neurosci. 2010 Feb;13(2):253-60. doi: 10.1038/nn.2466. See Fig. 1 and methods). Another example is Bajo et al. (Nat Commun. 2019 Jul 12;10(1):3075. doi: 10.1038/s41467-019-10770-4), where we tested sham-operated animals for 2 years in sound localization paradigms with and without earplugs and saw no degradation in their ability to adapt to the altered spatial cues resulting from unilateral earplugging over long periods of testing (see Bajo et al. 2019, Supplementary Figure 4, n=13). In addition, experimental cases with intracranial surgery under general anaesthesia to implant fibre optic devices for 30 months (n=13) and multiple periods of behavioural testing showed no changes in their health and consistently normal sound localization other than a specific impairment in earplug adaptation when the auditory cortex was optogenetic silenced (see Bajo et al., 2019, Supplementary Figure 5).

In the revised version of the manuscript, we added a paragraph to the methods to specify that while no specific controls have been used in this study, the controls and experimental groups used in several previous studies showed no indication of any alteration in behaviour due to cranial surgery under general anaesthesia or prolonged behavioural testing.

Line 109. “The longitudinal design of this study spanning more than 9 months allowed each animal to act as its own control by comparing the results before and after noise overexposure. We did not use specific controls comprising implanted animals without noise overexposure, since previous ferret experiments show no changes in behavioural testing or neural recordings following similar surgical procedures and/or intracranial implantation (e.g. Bajo et al., 2010; 2019). In addition, one separate, naïve animal was used as a control to compare physiological correlates of tinnitus with two out of the three ferrets implanted for chronic recordings (see methods).”

We have further strengthened our study by incorporating an additional physiological correlate of tinnitus. Specifically, we conducted recordings using Neuropixels probes in the auditory cortex of two out of three implanted ferrets, as well as in one sham-operated control for comparison. Initially, we had planned to present these data in a future publication; however, in response to your suggestion, we have now included them in the revised manuscript. These recordings were performed under general anaesthesia following the completion of the experimental protocol. Briefly, we found that both assessed NOE animals exhibited a significantly greater median spontaneous activity than units recorded in the control case (Kruskal-Wallis, χ^2^ _(2,1073)_ = 122.83; p<0.0001) (new supplementary Fig 16A), as well as shortened interspike intervals (ISIs). Although we cannot fully untangle the contribution of this change in excitability to the reduction of the ISI, it is worth noting that such shortening was more pronounced in control units than after NOE. When considering together the observed differences in spontaneous activity between cases and the less marked changes in the ISI, both are suggestive of a reduced temporal precision in the cortical activity of the NOE animals, which is consistent with the behavioural changes exhibited by those animals.

The methods and results for this experiment have been included in the main manuscript, and a new supplementary figure added (see below).

Line 480:

**Final recordings with Neuropixels probes in the Auditory Cortex**

In two out of the three implanted cases (Case 2 and Case 3) and in one additional control, we performed terminal recordings under general anaesthesia in the left auditory cortex (contralateral to the noise overexposured ear) using Neuropixels probes.

General anaesthesia was induced and maintained using Domitor (0.022 mg/kg/h) and Narketan 10 (5 mg/kg/h) in 0.9% sodium chloride, pH 7.2-7.4 with 5% glucose delivered intravenously (3-5 ml/h) for the complete duration of the recordings (24-36 hours). After tracheal intubation, the animal was artificially ventilated. Atropine sulphate (0.06 mg/kg s.c.), doxapram hydrochloride (4 mg/kg, s.c.), dexamethasone (0.5 mg/kg s.c.) and viscotears were administer every 6 hours to minimize pulmonary secretion, reduce respiratory depression, prevent cerebral oedema, and protect the eyes, respectively. Perioperative analgesia and antibiotics were also administered during the surgery as mentioned for the previous surgery, and depth of anaesthesia, respiratory rate, ECG, and end-tidal CO_2_ were monitored. Rectal temperature was maintaining at 38 °C with the help of a homeothermic electric blanket and a forced air-warming system.

After removing the chronic implant in the NOE animals, a craniotomy (4x4 mm^2^) was performed over the ectosylvian gyrus to reveal the auditory cortex. The animal was placed in an anechoic chamber and the *dura mater* was opened to visualize the primary auditory cortex.

Neural recordings were performed under anaesthesia (mixture of Domitor (medetomidine hydrochloride, 0.022 mg/kg, Orion Pharma; and Ketaset (ketamine hydrochloride, 5 mg/kg, Fort Dodge Animal Health) using Neuropixels 3A probes (single shank with 384 channels) and streamed to disk with SpikeGLX (<https://billkarsh.github.io/SpikeGLX/>) (sampling rate 25 kHz and filtered between 300 and 3000Hz). Spike clusters were identified using Kilosort2 spike sorting algorithm (Pachitariu et al 2016), curated by Phy (<https://github.com/kwikteam/phy>) and further analyzed using custom-written scripts in Matlab (R2023a). We analyzed the spontaneous firing behaviour of clusters that were responsive to broadband noise (duration 300ms, intensity 60-90 dB SPL), as defined by spike counts in the first 100 ms window (1ms bins) following the stimulus presentation being significantly different from a window of the same duration preceding the stimulus (paired t-tests, P<0.05).

Line 862:

**Neuropixels Recordings in the Auditory Cortex**

In two out of the three implanted cases (Case 2 and Case 3) and in one additional control, we performed final recordings in the left auditory cortex (contralateral to the noise overexposured ear). Acoustically-responsive cortical units recorded in NOE Cases 2 (median/interquartile 0.958/5.527) and 3 (md/iqt 2.713/5.764) exhibited a significantly greater median spontaneous activity than units recorded in the control case (md/iqt 0.275/1.975) (Kruskal-Wallis, χ^2^ _(2,1073)_ = 122.83; p<0.0001) (supplementary Fig 16A). The cortical units also had a broader distribution of firing rates after NOE than in the control animal (supplementary Fig 16B), indicating that the observed increase in median spontaneous firing rate was due to an overall increase with fewer units exhibiting a low spontaneous firing rate. Bursting activity was very low for all cases (control: 0.025 Hz, case 2: 0.012Hz and case 3: 0.004 Hz) with only 12.6% (30/237 units) in case 2, 5.4% (16/294 units) in case 3 and 9.6% (52/543 units) in the control case producing at least one burst of spikes.

To gain an insight into the temporal precision of the cortical responses, we calculated for each unit the change in the interspike intervals (ISI) during their evoked activity (100ms after stimulus presentation) compared to their spontaneous activity. Because we only analyzed acoustically-responsive units, the increased number of spikes would result in shorter ISI and therefore negative differences as was observed for all three cases, control and NOE (supplementary Fig 16C). Although we cannot fully untangle the contribution of the change in the excitability of the units to the reduction in the ISI, it is worth noting that such shortening was more pronounced in control units than after NOE. The observed differences in spontaneous activity between cases and less marked changes in the ISI are together suggestive of reduced temporal precision in the cortical activity in the NOE animals, which is consistent with the behavioural changes exhibited by those animals.

Line 1299:

**Fig S16. Increased cortical spontaneous activity after noise overexposure.** **(A)** Spontaneous activity of acoustically-responsive neurons for a control case (n= 543 units) and two NOE animals Case 2 (n=237) and Case 3 (n=294). NOE animals exhibited significantly higher spontaneous activity (Kruskal-Wallis, χ^2^ _(2,1073)_ = 122.83; p<0.0001) and a greater variability as indicated by the larger interquartile range. In each box, the central mark indicates the median, and the bottom and top edges of the box indicate the 25th and 75th percentiles, respectively. The whiskers extend to the most extreme data points not considered to be outliers, and the outliers are plotted individually using the '+' marker symbol. **(B)** Cumulative distribution of cortical units according to their spontaneous firing rates for the three cases. Cumulative functions for NOE cases showed a broader distribution skewed towards higher rates. **(C)** Difference between evoked (to broadband noise presentation) and spontaneous interspike intervals (ISI). Although a reduction of the ISI with stimulus presentation was expected because of increased activity following the stimulus, this reduction was significantly more marked in the control case than after NOE (Kruskal-Wallis, χ^2^ _(2,629)_ = 28.97; p<0.0001), suggesting that the temporal properties of the responses was less precise in NOE cases.

Therefore, in order to claim a link between tinnitus and sleep disruptions, the authors should provide an analysis of baseline variability due to real underlying variability and/or experimental noise. Another way to address this concern would be to point to other studies of ferret sleep architecture that may give a basis for comparison, if there are any. If these elements are not included, the authors should instead adjust their claims accordingly.

R1#3. Within each of the three cases, the variability in durations of wakefulness and NREM, REM and REM2 sleep were very similar across days. We now included standard errors across 2 days of baseline assessment of vigilance state durations in Fig. 2F to visualize it. In this study, ferrets spend 75.9±4.86% of time per day asleep, which matches the sleep duration of ferrets assessed in previous experiments (70.34 ± 1.69%, Jha et al 2006 and ‘above 60%’ in a preliminary study in two male ferrets by Marks & Shafferty 1996). The distribution of vigilance states also matched that of systematic previous reports, with female ferrets spending most of the time in NREM sleep (Jha et al: NREM = 43.67 ± 3.56, this study: 44.13±3.51), followed next by Wake (Jha et al: W = 29.60 ± 5.02, this study: 24.1±4.86), then REM sleep (Jha et al: REM = 19.3 ± 0.93; this study 16.63±4.52), the least in REM2 (Jha et al: REM-2 = 4.99 ± 2.25, this study: 10.67±2.78). We did not assess transitional phases as coined by Jha et al (2006) due to their relatively vague definition but focused on consolidated vigilance states. While there are some procedural differences between this study and Jha et al (2006) (e.g. a 12/12 light dark cycle vs a 15/9 light dark cycle in this study), as well as analytical differences (Jha et al used an 8s window for the vigilance scoring procure, this study used a 4s window), the similarity in amount of wakefulness and sleep, distribution of vigilance states and variability between individuals suggests consistent ferret sleep-wake behaviour across studies.

The following has been added to the manuscript:

Line 655: The animals spent most of the time (75.9±4.86%) sleeping in undisturbed baseline conditions (71.5%, 85.6%, and 70.6%, Fig. 6A–C), similar to previously reported sleep durations in ferrets (70.34±1.69%, [41]). Durations in vigilance state durations were similar across days in each ferret (Fig. 2F) and variability between animals also closely matched previous reports [41]).

Other comments and questions listed below:
1. Was the position of the ferret taken into consideration when presenting stimuli during sleep? In other words, how was an even sound field constructed so that the SPL would not vary at different locations in the chamber? I imagine at lower frequencies this would not be much of an issue, but perhaps at 16kHz, for example, there could be large differences in SPL at different locations in the chamber.

R1#4. We understand reviewer’s concern but the position of the ferret barely changed from the centre of the enclosure when it sleeps since that is where the bedding material was placed. As stated in the Methods, the auditory stimuli were presented via a free–field loudspeaker installed on the ceiling of the double–walled, sound–attenuated recording chamber above the custom–made enclosure. Stimuli were presented at 40, 50, 60 and 65 dB SPL (as measured at floor level at the centre of the enclosure).

We have added this information in the same paragraph (Lines 437-440), which now reads:

“In the first 24 h of each recording session, the animal was left undisturbed. Over the course of the second 24 h of the session, auditory stimuli were presented via a free–field loudspeaker installed on the ceiling of the double–walled sound–attenuated recording chamber above the custom–made enclosure. Sounds were one octave narrowband stimuli with centre frequencies of 1, 4, 8 and 16 kHz. Stimuli had a duration of 820 ms and included a silent gap of 38 ms in the middle of the stimulus. Stimuli were presented at 40, 50, 60 and 65 dB SPL (as measured at floor level at the centre of the enclosure, where the bedding material in which the ferret sleeps was located).”

2. Related to the first point, why was unilateral NOE used, and if animals slept with one ear down by the floor, was there a requirement that the exposed versus intact ear would be facing the speaker?

R1#5.

We thank the reviewer for pointing out this need for clarification. Unilateral NOE is the standard for tinnitus animal models to prevent hearing loss due to bilateral exposure (for example Dehmel et al. (Journal of Neuroscience 2012, 32 (5) 1660-1671) in guinea pigs, Bauer & Brozolski (2001) in rats, or Brozoski et al. (2002) in chinchilla. Hence, we used a similar approach for our study. We added this information to the manuscript as follows:

Line 193: “In line with previous work in which noise exposure was used to induce tinnitus in animal models, we the noise was applied unilaterally in order to prevent hearing loss in both ears [45, 46, 47].”

The loudspeaker was installed on the ceiling of the recording chamber and intensity levels were measured at floor level at the centre of the enclosure. It was not a requirement that the intact ear faced the speaker. However, the ferrets commonly changed sleeping postures multiple times during any given dark period, which included positions exposing the left or the right ear, or both ears evenly if the animal curled up with the head in a horizontal position (see examples below). Because of this we decided to present stimuli over 24 hours and in a randomized sequence throughout this period, minimizing the chance of favouring the exposure of a particular ear.

3. The tinnitus index calculation is a bit confusing-- 1/3 of the index is based on performance at detecting a continuous sound, which is added to the other metrics and not weighed differently. This seems to be a readout of behavioral performance and hearing sensitivity, rather than tinnitus per se. Why is it added to the other values rather than used to normalize or otherwise scale the values?

R1#6.

We thank the reviewer for pointing this out. We used a metric for the animal’s ability to detect continuous sounds based on the assumption that an animal with tinnitus is unlikely to only show an impairment in gap detection ability. In addition, and unlike an animal with hearing loss alone, an animal experiencing a continuous phantom sound (tinnitus) should also make fewer errors in detecting continuous sounds due to this constant percept. Hence, we posit that the ability to detect continuous sounds can additionally serve as a metric for tinnitus.

Importantly, to assess the impact of hearing loss on behaviour in both the silent gap detection paradigm and the silence detection paradigm, we assessed the auditory brainstem response in all ferrets. We decided to present the degree of hearing loss based on this readout separately from the behavioural tinnitus index, as animals may experience, just like humans, a combination of both tinnitus and hearing loss.

We added the following section to the manuscript:

Line 357: “The metric for operant gap detection performance refers to the change in ability to detect the continuous sound (no gap) across all tested stimulus frequencies (Eq(2)) and to the change in gap–detection threshold across all stimuli (Eq(3)). Eq(2) is based on the assumption that an animal experiencing a continuous phantom sound (tinnitus) should also make fewer errors in detecting continuous sounds due to this constant percept and therefore show improved continuous sound detection ability as compared to before the tinnitus trigger.”

In addition, the validity of the behavioural tinnitus indices has been reinforced by adding physiological correlates of tinnitus (see response to Reviewer 2, R2#2).

4. The baseline tinnitus index (pre-exposure) should be shown in figure 3A.

R1#7. The tinnitus index as shown in Fig.3A reflects differentials in behavioural metrics between baseline and post NOE assessments and can therefore not be shown separately for baseline conditions. However, to illustrate tinnitus-sensitive behaviour under baseline conditions as well as after noise exposure, we produced additional panels (Fig.3B) showing absolute values for gap detection ability in baseline conditions (pre-exposure) and after exposure for all three cases.

The updated Fig.3 and legend (revised accordingly) have now been included in the main manuscript.

Line 1096: “**Fig 3.** **Tinnitus development, hearing loss and sleep disruptions assessed over time.** (**A**) Tinnitus and hearing loss development over time in three different ferrets (Ferrets 1-3). Yellow plots depict behavioural evidence for tinnitus (using the tinnitus index, TI, for each animal (based on operant gap and silence detection; see Methods for details)). Light grey bars show hearing loss based on the differences from baseline in ABR thresholds while dark grey bars show the change in ABR total RMS magnitude, respectively. The bars are a depiction of the metrics for TI and ABR changes displayed in Figure S3. The size of each bar indicates the change relative to BL of the corresponding measure at Post 1 and Post 2. Each panel represents a different ferret. Light red/light brown shading for Ferret 1 highlight measurements conducted in Post 1 (1 week) and Post 2 (6 months). Plots for Ferrets 2 and 3 have identical layout. Panels left to right: Case 1, Case 2, Case 3. (**B**) Impairment in gap detection ability over time based on thresholds. Displayed values correspond to the m(thresh) metric used to calculate the tinnitus index (TI, see panel A) for each individual case. Values are based on gap detection thresholds fitted on a continuous scale ranging from 0-100%. 100% corresponds to a gap detection threshold of 3ms, and the remaining gap lengths (5, 10, 20, 50, 270ms) correspond to the evenly spaced decrements in threshold (see methods for details). Note that an increase in gap detection threshold over time is represented by a decrease in the values presented here. (**C**) Number of wake episodes during ~48 hours of baseline recording. Large panels are histograms depicting the number of wake episodes separated by episode duration and the insets are the total number of wake episodes for baseline (BL), one week (Post 1) and 6 months (Post 2). The y-axis and the number displayed in the bars in the inset panels depict the number of wake episodes. Note the difference in y-axis scale for ferret 3. Panels left to right: Ferret 1, Ferret 2, Ferret 3. Note that in Ferret 2 the number of wake episodes of nearly all durations increase six months following NOE (Post 2) and overlap the other bars (Baseline and one week post NOE, Post 1) in the panel.”

5. The EEG recordings, at least in case 2 & 3, appear to degrade significantly in terms of quality by the 6 month time point. How do the authors verify the accuracy of their sleep stage scoring results given the increase in noise?

R1#8. In preparation of the vigilance scoring procedures, EEG data were routinely band-path filtered, excluding frequencies above 30Hz and below 0.5 Hz, which removed high frequency noise present in the raw data. Vigilance state scoring relied on modulation in low frequency bands (NREM 0.5-4Hz, REM 4.5-8Hz, REM2 8.5-20Hz), which were largely unaffected by noise. Despite some degradation of signals over time, vigilance states could be unequivocally determined.

A clarification was included in the main manuscript as follows:

Line 406: “Vigilance state scoring was performed manually offline prior to spectral analysis and assessment of sleep architecture. For data visualisation the EEG was additionally filtered between 0.5 and 30 Hz, and the EMG between 10 and 49 Hz with an additional 50 Hz notch filter to ensure attenuation of potential electrical current noise.

6. The discussion could be enhanced by discussing the findings in humans with tinnitus, as a few polysomnography studies have indicated increased sleep latency and decreased deep NREM and REM as potentially linked with the condition. This could serve as a point of comparison to the ferret results.

R1#9. Following the Reviewer’s suggestion, we have added a paragraph in the discussion comparing human and ferret results.

Line 920: “We obtained chronic EEG recordings to investigate changes in evoked activity and sleep-wake pattern in parallel to emerging tinnitus and hearing impairment. Interestingly, we found changes that are common in the three cases and have been previously reported in humans with tinnitus. REM2 sleep amount increased in the three cases followed noise overexposure (Week 1) regardless of their different initial magnitude of tinnitus, although only transiently for ferrets 1 and 3 (Fig S4). This may suggest that brain activity characteristic for this state, in particular oscillations in the beta and possibly gamma range, may be a marker for consequences of NOE. In humans exhibiting residual inhibition, gamma activity is positively correlated with tinnitus [73]. Although not many sleep studies had been performed in humans with chronic subjective tinnitus, polysomnography studies revealed pathological sleep patterns, with tinnitus patients remaining longer in shallow sleep (stages 1 and 2) and spending less time in deep sleep (stage 3) and REM sleep [Eysel-Gosepath & Selivanova (2005); Hébert et al. (2011); Attanasio et al. (2013). Those differences were not always statistically significant [Chang et al., 2024] but were more evident in those patients where tinnitus was highly modulated by sleep [Guillard et al., 2023], suggesting that tinnitus heterogeneity is always a factor to consider, both in human and animal studies. Interestingly, lower spectra power in the delta frequency band that correlates with subjective sleep complaints has been reported in humans [Hébert et al. (2011)].”

**Reviewer #2**: General
This study attempts first to develop a new model of tinnitus in ferrets, and second to study the effects of sleep. These would be better separated into two papers, enabling the authors to more thoroughly develop the model and establish its validity before going on to study the effects of sleep,

R2#1. We thank the Editor and Reviewer for the suggestion to separate this study into two papers, the first one about the ferret as animal model of tinnitus, and the second one studying the interaction between tinnitus and sleep. We know that we have strong data for the first part of our study and only representative cases for the second. However, we believe that the three case studies serve as valuable pilot data to motivate and guide further study of sleep and tinnitus in the ferret model. Importantly, the aim of this study was not only to present the ferret as a model to study chronic tinnitus, but also as one to specifically study tinnitus in the context of sleep. We acknowledge that the cases presented for the latter part of the study do not offer a sample size large enough to draw general conclusions.

Nevertheless, our findings of sleep alterations and changes in brain activity following induced tinnitus reflect part of the phenotype observed in human tinnitus sufferers. This provides further evidence that our ferret model of this condition, which is notoriously difficult to study, is robust and also highly promising for studying the relationship between sleep and tinnitus. Additionally, we present valuable insights into this relationship, which has not been extensively investigated but offers remarkable potential for progress in the tinnitus field.

Therefore, we think that it is most beneficial for the tinnitus and sleep fields if both parts of the manuscript are published together, and in a timely manner.

See also later comment in (R2#13).

In the first part, while ambitions and meritorious in its scope of having three separate behavioral measurements of tinnitus, there are some factors that decrease enthusiasm for the application of these methods. First, only a percentage of animals (and people) develop tinnitus after noise over exposure. Thus, it would be important to show individual data for each animal rather than just mean results. Second, correlating the behavior with a physiological correlate of tinnitus (eg increased spontaneous rates or synchrony in an auditory structure) would add to the validity of these behavioral measures.

R2#2. We agree with the reviewer that it is important to show individual data for each animal because only a percentage of animals develop tinnitus after noise overexposure. Behavioural data in Fig. 1 and Supplementary Figure S1 and S2, show individual data. There were two exceptions (panel B and G from Fig. 1) where, for clarity we used only the means and SD. In the revised version, we have changed those panels

(operant gap detection thresholds and ABR thresholds per stimulus type) and individual data have been added.

The validity of the behavioural tinnitus indices has been reinforced by adding physiological correlates of tinnitus derived from new recordings from the auditory cortex (see for details response to Reviewer 1 (R1#2).

Enthusiasm for the results of the study is diminished considering the progressive increase in ABR thresholds observed over the 6 months. This is problematic as it is now difficult to really distinguish the effects of hearing loss (threshold shifts) and tinnitus. It is surprising that there is a progressive threshold increase in ferrets at 6 months as other studies in animals have shown temporary threshold shifts with this level of noise exposure delivered under anesthesia.

R2#3. We used the same settings for noise overexposure validated in other animal models, mainly rodents, and we expected, as the Reviewer points out, to have similar temporal threshold shifts. However, this was not the case for ferrets, and we think that it is important to communicate this as part of introducing a new animal model. Future experiments in ferrets should modify the NOE paradigm, possibly deviating from that used in other animal models, to produce behavioural impairment suggestive of tinnitus without hearing loss. Interestingly, in our study, individual cases show that tinnitus and hearing loss did not develop equally in all three cases following the same peripheral insult (see Fig 3A). We believe it is important to communicate this as well, as a first step towards assessing whether tinnitus heterogeneity in the ferret model reflects that seen in human tinnitus suffers. We have added a paragraph in the discussion about this limitation (hearing loss and tinnitus) and future development.

Line 964:

“The main limitations from this case study are the absence of temporary shifts in ABRs following noise overexposure, the small number of animals with chronic implants, and the absence of specific ad hoc controls. The relationship between hearing loss and tinnitus has long been recognised (e.g. Axelsson and Sandh, 1985, reviewed in Gold and Bajo, 2014), even when only a hidden hearing loss is present [Schaette and McAlpine, 2011]. Future studies varying the parameters used for noise overexposure will decipher whether it is possible to disentangle hearing loss and tinnitus in the ferret model and its respective interaction with sleep states. This study is a case study utilizing data from only three individual cases to explore the relationship between tinnitus and sleep and with no specific control group. However, its validity is reinforced by its longitudinal design and the fact that baseline sleep variability in our cases aligns with previously described patterns in ferrets. Moreover, our previous studies have shown that similar experimental procedures (intracranial surgery and chronic implants) to the ones used here do not produce behavioural or neuropathological changes (Bajo et al., 2010, 2019), indicating that the changes that we observed were most likely caused by the NOE procedure, further supporting our methodology. Future work with increased animal numbers and longitudinal controls will be needed to build on the model and approach we introduce in this study and systematically address the relationship between sleep and tinnitus suggested by our initial data.”

The idea of examining the relationship of tinnitus to sleep is interesting. Strengthening the theoretical link at the outset would improve the impact of the paper. The same limitations would apply to this second part of the paper as hearing loss would likewise affect the interpretation of tinnitus.

R2#4. This is a very valuable suggestion, and we revised the manuscript accordingly, by mentioning the hypothesised relationship between tinnitus and sleep in the abstract (see response to R2#5) but see also lines 63-76 of the Introduction.

Specific
Abstract:
Rationale for exploring sleep and tinnitus not made clear in abstract – should make this clearer.

R2#5. We have modified the abstract to make the rational for exploring sleep and tinnitus more explicit.

Line 20-23. “Subjective tinnitus is a phantom auditory perception in the absence of an actual acoustic stimulus that affects 15% of the global population. In humans, tinnitus is often associated with disturbed sleep and, interestingly, there is an overlap of the brain areas involved in tinnitus and NREM sleep. We used…”

Rather state no available cure as there are effective treatments in recent developments.

R2#6. We have deleted “no available cure” in the abstract (line 22).

Introduction
First paragraph is very complex and difficult to follow. Suggest simplifying.

R2#6. The first paragraph in the Introduction has been simplified for clarity.

Lines 48-57:

“Subjective tinnitus, or tinnitus for short, is the most prevalent phantom sensation [1, 2]. It is commonly reported as the constant perception of a hissing, ringing or buzzing sound without any identifiable source [1] and is often associated with disturbed sleep [3-11]. During sleep, the brain is relatively disconnected from the external environment [12-14], neural activity is mainly generated endogenously, and phantom percepts during dreams are considered normal. In contrast, phantom percepts during wakefulness can be a reflection of neuronal malfunction ([15]; work of Ambroise Paré reviewed in 16-18]. Despite the association between sleep and tinnitus, very little is known about the relationship between the neuronal changes that take place during sleep and those that give rise to persistent phantom percepts.”

Second paragraph provides a different rationale and aim for the study – ie to develop a new animal model for tinnitus. Perhaps soften the wording here – eg use a different model to study sleep-tinnitus interactions.

R2#7. The sentence has been changed to state that the main aim was to address this interaction between sleep and tinnitus by using a new animal model.

Line 75-78: “The main aim of this study was twofold: first, to introduce a new animal model for assessing the interaction between sleep and chronic tinnitus, and to validate this model by examining sleep alterations following tinnitus induction; and second, to gather preliminary data to empirically test for a bidirectional relationship between tinnitus and sleep. The ferret…”

Methods
Only 3 animals were used for chronic recordings. It would be good to have more animals with and without tinnitus without the confound of hearing loss. Consider developing the model to produce a temporary theshold shift instead of permanent.

R2#8. As mentioned in our response to the Editor and Reviewer 1 (E#1, R1#2&3), one of the primary limitations of our study is the small sample size, with chronic recordings conducted on only three ferrets. This limitation is why our work is characterized as "A case study," as indicated in the title of the paper.

The aim of this study was to set up a new animal model for chronic tinnitus and for researching its relationship with sleep. We have described three individual cases that provide first evidence for a bi-directional interaction between tinnitus and natural brain dynamics and our data suggest that different sleep patterns may be related to different degrees of tinnitus and/or hearing loss. We believe that, despite its inherent limitations as a case study, this work makes a significant contribution to the field of tinnitus research. For the first time, it proposes, tests, and employs an empirical approach to systematically investigate the connection between chronic tinnitus and sleep. This study lays the groundwork for a novel research perspective that is crucial for advancing our understanding of tinnitus and potentially developing new treatment strategies.

It is important to stress that our data reveal important inter-individual differences in hearing loss and tinnitus between animals, as is also known to be the case in patients. The variability in tinnitus cases observed in this study suggests that increasing the number of animals and averaging the data may dilute and potentially mask important individual profiles. We certainly acknowledge that having a larger sample size would be desirable, but the priority here was to carry out a longitudinal investigation using a comprehensive combination of behavioural and electrophysiological measures. It took almost two years to obtain these data from each animal, so it is not feasible to add any more to this study. We certainly agree that future work should consider developing the model further to produce temporary instead of permanent threshold shifts.

As mentioned, one of the unique benefits of the ferret model (compared to rodents) is that we were able to conduct chronic recordings in a longitudinal design, allowing each animal to be its own control. To increase the validity of the longitudinal study, in the revised version, we have analysed in the baseline variability in sleep architecture as suggested by reviewer #1 (R1#3) and we show that our ferrets have similar sleep-wake distribution as shown in previous studies that investigated sleep in this species.

The following has been added to the manuscript:

Line 655: The animals spent most of the time (75.9±4.86%) sleeping in undisturbed baseline conditions (71.5%, 85.6%, and 70.6%, Fig. 6A–C), similar to previously reported sleep durations in ferrets (70.34±1.69%, [43]). Durations in vigilance state were similar across days in each ferret (Fig. 2F) and variability between animals closely matched previous reports [43].

Tinnitus measurements – three behavioral measurements is a strength. However, previous gap detection paradigms were of highest value when showing correlations with physiological states that indicate tinnitus (eg increased spontaneous rates and synchrony of an auditory region). Is there some activity that could serve this purpose in the present study?

R2#9. This is a very good point. The validity of the behavioural tinnitus indices has been reinforced by adding physiological correlates of tinnitus (see for details response to Reviewer 1 (R1#2).

We have further strengthened our study by adding new electrophysiological data to the paper which provide the neural correlates of tinnitus that were requested by the reviewer. Specifically, we now analysed recordings made using Neuropixels probes in the auditory cortex of two out of three implanted ferrets, as well as in one sham control for comparison. These recordings were performed under general anaesthesia following the completion of the experimental protocol. Briefly, we found that both assessed NOE animals exhibited a significantly greater median spontaneous activity than units recorded in the control case (Kruskal-Wallis, χ^2^ _(2,1073)_ = 122.83; p<0.0001) (new supplementary Fig 16A) as well as shortened interspike intervals (ISIs). Although we cannot fully untangle the contribution of the change in excitability to the reduction in the ISI, it is worth noting that such shortening was more pronounced in control units than NOE. The observed differences in spontaneous activity between cases and the less marked changes in the ISI together suggest that cortical activity may show reduced temporal precision in the NOE animals, which is congruent with the behavioural changes exhibited by those animals.

The methods for this experiment have been included in the main manuscript, results have been added, and a new supplementary figure (see below).

Line 480:

**Final recordings with Neuropixels probes in the Auditory Cortex**

In two out of the three implanted cases (Case 2 and Case 3) and in one additional control, we performed terminal recordings under general anaesthesia in the left auditory cortex (contralateral to the noise overexposured ear) using Neuropixel probes.

General anaesthesia was induced and maintained using Domitor (0.022 mg/kg/h) and Narketan 10 (5 mg/kg/h) in 0.9% sodium chloride, pH 7.2-7.4 with 5% glucose delivered intravenously (3-5 ml/h) for the complete duration of the recordings (24-36 hours). After tracheal intubation, the animal was artificially ventilated. Atropine sulphate (0.06 mg/kg s.c.), doxapram hydrochloride (4 mg/kg, s.c.), dexamethasone (0.5 mg/kg s.c.), and viscotears were administer every 6 hours to minimize pulmonary secretion, reduce respiratory depression, prevent cerebral oedema, and protect the eyes, respectively. Perioperative analgesia and antibiotics were also administered during the surgery as mentioned for the previous surgery, and depth of anaesthesia, respiratory rate, ECG, and end-tidal CO_2_ were monitored. Rectal temperature was maintaining at 38 °C with the help of a homeothermic electric blanket and a forced air-warming system.

After removing the chronic implant in the NOE animals a craniotomy (4x4 mm^2^) was performed over the ectosylvian gyrus to reveal the auditory cortex. The animal was placed in an anechoic chamber and the *dura mater* was open to visualize the primary auditory cortex.

Neural recordings were performed under anaesthesia (mixture of Domitor (medetomidine hydrochloride, 0.022 mg/kg, Orion Pharma; and Ketaset (ketamine hydrochloride, 5 mg/kg, Fort Dodge Animal Health) using Neuropixels 3A probes (single shank awith 384 channels) and streamed to disk with SpikeGLX (<https://billkarsh.github.io/SpikeGLX/>) (sampling rate 25 kHz and filtered between 300 and 3000Hz). Spike clusters were identified using Kilosort2 spike sorting algorithm (Pachitariu et al 2016), curated by Phy (<https://github.com/kwikteam/phy>) and further analyzed using custom-written scripts in Matlab (R2023a). We analyzed the spontaneous firing behaviour of clusters that were responsive to broadband noise (duration 300ms, intensity 60-90 dB SPL), as defined by spike counts in the first 100 ms window (1ms bins) following the stimulus presentation being were significantly different from a window of the same duration preceding the stimulus (paired t-tests, P<0.05).

Line 862:

**Neuropixels Recordings in the Auditory Cortex**

In two out of the three implanted cases (Case 2 and Case 3) and in one additional control, we performed final recordings in the left auditory cortex (contralateral to the noise overexposured ear). Acoustically-responsive cortical units recorded in NOE Cases 2 (median/interquartile 0.958/5.527) and 3 (md/iqt 2.713/5.764) exhibited a significantly greater median spontaneous activity than units recorded in the control case (md/iqt 0.275/1.975) (Kruskal-Wallis, χ^2^ _(2,1073)_ = 122.83; p<0.0001) (supplementary Fig 16A). The cortical units also had a broader distribution of firing rates after NOE than in the control animal (supplementary Fig 16B), indicating that the observed increase in median spontaneous firing rate was due to an overall increase with fewer units exhibiting a low spontaneous firing rate. Bursting activity was very low for all cases (control: 0.025 Hz, case 2: 0.012Hz and case 3: 0.004 Hz) with only 12.6% (30/237 units) in case 2, 5.4% (16/294 units) in case 3 and 9.6% (52/543 units) in the control case producing at least one burst of spikes.

To gain an insight into the temporal precision of the cortical responses we calculated for each unit the change in the interspike intervals (ISI) during their evoked activity (100ms after stimulus presentation) compared to their spontaneous activity. Because we only analyzed acoustically-responsive units, the increased number of spikes would result in shorter ISI and therefore negative differences as was observed for all three cases, control and NOE (supplementary Fig 16C). Although we cannot fully untangle the contribution of the change in the excitability of the units to the reduction in the ISI, it is worth noting that such shortening was more pronounced in control units than after NOE. The observed differences in spontaneous activity between cases and less marked changes in the ISI are together suggestive of reduced temporal precision in the cortical activity in the NOE animals, which is consistent with the behavioural changes exhibited by those animals.

Line 1299:

**Fig S16. Increased cortical spontaneous activity after noise overexposure.** **(A)** Spontaneous activity of acoustically-responsive neurons for a control case (n= 543 units) and two NOE animals Case 2 (n=237) and Case 3 (n=294). NOE animals exhibited significantly higher spontaneous activity (Kruskal-Wallis, χ^2^ _(2,1073)_ = 122.83; p<0.0001) and a greater variability as indicated by the larger interquartile range. In each box, the central mark indicates the median, and the bottom and top edges of the box indicate the 25th and 75th percentiles, respectively. The whiskers extend to the most extreme data points not considered to be outliers, and the outliers are plotted individually using the '+' marker symbol. **(B)** Cumulative distribution of cortical units according to their spontaneous firing rates for the three cases. Cumulative functions for NOE cases showed a broader distribution skewed towards higher rates. **(C)** Difference between evoked (to broadband noise presentation) and spontaneous interspike intervals (ISI). Although a reduction of the ISI with stimulus presentation was expected because of increased activity following the stimulus, this reduction was significantly more marked in the control case than after NOE (Kruskal-Wallis, χ^2^ _(2,629)_ = 28.97; p<0.0001), suggesting that the temporal properties of the responses was less precise in NOE cases.

Please provide more rationale for assessing ‘vigilance state’ from frontal and occipital regions.

R2#10. Assessing frontal and occipital EEG derivations for vigilance state scoring is a widely used approach in rodent models for sleep research [36,45,46]. This method has also been successfully applied in seminal studies of sleep in ferrets (Marks & Shaffery 1996; Jha et al 2006). Frontal EEG derivation is optimally positioned to record frontal cortical slow wave activity, the hallmark of NREM sleep, and the occipital derivation is optimally positioned to pick up activity in the theta range, the hallmark of REM sleep. Therefore, we opted for a similar approach in our ferret model. We did not previously specify in the manuscript that the approach of using frontal and occipital EEG derivations for sleep scoring was based on previous studies, but clarified this as follows:

Lines 177-180 was modified to address this concern: “Similar configurations using frontal and occipital EEG electrodes, optimal for recording cortical slow-wave and theta activity, have been used to provide recordings suitable for vigilance state scoring across different mammalian species such as ferrets, rats and mice [36,45,46].”

It is stated that silent gap detection decreased after noise exposure. The results show mean increases in gap detection. This is problematic that individual animals are not shown as previous studies have shown that only about half of animals develop tinnitus, therefore it is not valid to show mean data. It would be better to separate the animals into those that develop tinnitus and compare them with those that do not develop tinnitus. The same is true fo the ABR results. Individual animals should be shown in addition to mean data.

R2#11. We agree. Panels B and G from Figure 1 have been changed to show individual data (already addressed in R2#2). Since we did not observe a clear bimodal distribution, we decided to not allocate animals into ‘tinnitus’ and ‘no tinnitus’ groups. Rather, we acknowledge that behavioural symptoms can lie on a continuum and conclude that the shift in behavioural performance across individuals is generally in line with tinnitus development. The longitudinal assessment in three cases for the second part of our study further shows differing degrees of behavioural symptoms indicative of tinnitus and with and without pronounced hearing loss.

In addition, it is problematic that the ABR thresholds increase progressively at one week and 6 months (unless the figure is incorrect). It would be helpful if the colors for 6 months and one week were more distinctive. But assuming the figure is correct, this means that the animals had hearing deficits of more than 40 dB at some frequencies. This also means that some of the gap detection results could be due to hearing loss and not to tinnitus. It is also unusual for animals to show worsening of ABR thresholds at 6 months for this level of noise exposure. Usually this produces a temporary threshold shift and not a permanent threshold shift as in this case.

R2#12. We apologize for the color choice in Fig.1G, which has now been revised (6 months corresponds now to a darker hue, making it more distinct from the ‘1 week’ plot). The ABR thresholds increase from ‘1 week’ to ‘6 months’ post NOE assessments, and ABR magnitude also decreases in two out of three implanted cases (dark grey bars in Figure 3A). The chronic cases present different degrees of tinnitus and hearing loss, whereas the animal with the least symptoms of hearing loss shows the strongest behavioural evidence for tinnitus (and vice versa). This heterogeneity in tinnitus and hearing loss across ferrets after identical noise exposure is an expected outcome, given the heterogeneity known to exist in human tinnitus sufferers.

As previously mentioned (see R2#3) we used the same settings for noise overexposure validated in other animal models, mainly rodents, and we expected to see temporary threshold shifts. The fact that the ferrets studied long-term in this study showed overall progressively increasing hearing loss until 6 months after NOE is an interesting observation and should be communicated. Future studies are needed to assess the appropriate NOE settings to induce reliable temporary threshold changes in the ferret model. We have added a paragraph to the discussion about this limitation (hearing loss and tinnitus) and future development.

Line 964-981:

“The main limitations from this case study are the absence of temporary shifts in ABRs following noise overexposure, the small number of animals with chronic implants, and the absence of specific *ad hoc* controls. The relationship between hearing loss and tinnitus has long been recognised (e.g. Axelsson and Sandh, 1985, reviewed in Gold and Bajo, 2014), even when only a hidden hearing loss is present [Schaette and McAlpine, 2011]. Future studies varying the parameters used for noise overexposure will decipher whether it is possible to disentangle hearing loss and tinnitus in the ferret model and its respective interaction with sleep states. This study is a case study utilizing data from only three individual cases to explore the relationship between tinnitus and sleep and with no specific control group. However, its validity is reinforced by its longitudinal design and the fact that baseline sleep variability in our cases aligns with previously described patterns in ferrets. Moreover, our previous studies have shown that similar experimental procedures (intracranial surgery and chronic implants) to the ones used here do not produce behavioural or neuropathological changes (Bajo et al., 2010, 2019), indicating that the changes that we observed were most likely caused by the NOE procedure, further supporting our methodology. Future work with increased animal numbers and longitudinal controls will be needed to build on the model and approach we introduce in this study and systematically address the relationship between sleep and tinnitus suggested by our initial data.”

Discussion
As the study is very long, it is suggested to divide this paper into two papers with the tinnitus model being the first paper. The discussion is very long and rambling and would be better focused specifically on the results of this study rather than numerous speculations regarding etiology, hyperacusis and other models.

R2#13. We thank the Reviewer for suggesting this alternative. As mentioned in response to the first comment, we believe that the study in its current form is warranted. It offers a significant advancement for the tinnitus field by presenting both, a model for the specific study of the interaction between chronic tinnitus and sleep, and foundational data on this relationship.

We agree with the reviewer that the discussion is very long and rambling. In the revised version, we have shortened the discussion and made it easier to read (Lines 888-1009).

6. PLOS authors have the option to publish the peer review history of their article ([what does this mean?](https://journals.plos.org/plosone/s/editorial-and-peer-review-process#loc-peer-review-history)). If published, this will include your full peer review and any attached files.

If you choose “no”, your identity will remain anonymous but your review may still be made public.

**Do you want your identity to be public for this peer review?** For information about this choice, including consent withdrawal, please see our [Privacy Policy](https://www.plos.org/privacy-policy).

Reviewer #1: No

Reviewer #2: No

[NOTE: If reviewer comments were submitted as an attachment file, they will be attached to this email and accessible via the submission site. Please log into your account, locate the manuscript record, and check for the action link "View Attachments". If this link does not appear, there are no attachment files.]

While revising your submission, please upload your figure files to the Preflight Analysis and Conversion Engine (PACE) digital diagnostic tool, <https://pacev2.apexcovantage.com/>. PACE helps ensure that figures meet PLOS requirements. To use PACE, you must first register as a user. Registration is free. Then, login and navigate to the UPLOAD tab, where you will find detailed instructions on how to use the tool. If you encounter any issues or have any questions when using PACE, please email PLOS at [figures@plos.org](mailto:figures@plos.org). Please note that Supporting Information files do not need this step.
