# Supplementary material for: Cortical evoked activity is modulated by the sleep state in a ferret model of tinnitus. A cross-case study
Source: PLoS One. 2024 Dec 4;19(12):e0304306. doi: 10.1371/journal.pone.0304306 (PMC11616861; doi:10.1371/journal.pone.0304306)
Supplement: Attachment — Submitted filename: Rebuttal Letter Revision 2.docx [file pone.0304306.s017.docx]

[Note: HTML markup is below. Please do not edit.]

Reviewers' comments:

Reviewer's Responses to Questions

**Comments to the Author**

1. If the authors have adequately addressed your comments raised in a previous round of review and you feel that this manuscript is now acceptable for publication, you may indicate that here to bypass the “Comments to the Author” section, enter your conflict of interest statement in the “Confidential to Editor” section, and submit your "Accept" recommendation.

Reviewer #1: (No Response)

Reviewer #3: (No Response)

Reviewer #4: (No Response)

2. Is the manuscript technically sound, and do the data support the conclusions?

The manuscript must describe a technically sound piece of scientific research with data that supports the conclusions. Experiments must have been conducted rigorously, with appropriate controls, replication, and sample sizes. The conclusions must be drawn appropriately based on the data presented.

Reviewer #1: Yes

Reviewer #3: Yes

Reviewer #4: Partly

3. Has the statistical analysis been performed appropriately and rigorously?

Reviewer #1: N/A

Reviewer #3: Yes

Reviewer #4: Yes

4. Have the authors made all data underlying the findings in their manuscript fully available?

The [PLOS Data policy](http://c05y1x9s.r.us-east-2.awstrack.me/L0/http:%2F%2Fwww.plosone.org%2Fstatic%2Fpolicies.action%23sharing/1/010f019203314c6f-c923db4e-3290-458c-baba-e29c59bbc5f6-000000/zZdxKrWaod3RKQuELZZD3Ee7N2I=177) requires authors to make all data underlying the findings described in their manuscript fully available without restriction, with rare exception (please refer to the Data Availability Statement in the manuscript PDF file). The data should be provided as part of the manuscript or its supporting information, or deposited to a public repository. For example, in addition to summary statistics, the data points behind means, medians and variance measures should be available. If there are restrictions on publicly sharing data—e.g. participant privacy or use of data from a third party—those must be specified.

Reviewer #1: Yes

Reviewer #3: Yes

Reviewer #4: Yes

5. Is the manuscript presented in an intelligible fashion and written in standard English?

PLOS ONE does not copyedit accepted manuscripts, so the language in submitted articles must be clear, correct, and unambiguous. Any typographical or grammatical errors should be corrected at revision, so please note any specific errors here.

Reviewer #1: Yes

Reviewer #3: Yes

Reviewer #4: Yes

6. Review Comments to the Author

Please use the space provided to explain your answers to the questions above. You may also include additional comments for the author, including concerns about dual publication, research ethics, or publication ethics. (Please upload your review as an attachment if it exceeds 20,000 characters)

**Reviewer #1:** I thank the authors for addressing my comments and adding valuable data to the manuscript as well as editing the text for clarity. I have a few remaining suggestions, the overall comment remaining being that the manuscript would benefit greatly from another round of editing on the figures.

-The manuscript can be hard to digest given that it explores the broad topics of tinnitus and sleep, and as a case study requires the reader to consider each individual rather than a big-picture result. As the authors chose to keep the paper together rather than splitting it as R2 suggested, to improve readability I would suggest moving a couple items from the supplemental material to the main figures:

-One of the main results highlighted in the discussion, that is consistent across the 3 animals -- an increase in REM2-- is not explicitly shown in a figure (the REM2 amounts are shown in separate figures for the baseline and post-NOE conditions). Pre- and post- NOE REM2 measurements (along with the amounts of other sleep stages) should instead be side-by-side in a figure, with error bars, and statistical analysis if possible.

Rev #1.1. We have added a new Fig. 5, which includes the panels from the original Fig. S5 along with additional panels (Fig. 5E) that explicitly show the average changes in REM2 over time (baseline, 1 week, and 6 months, presented side-by-side), as well as changes in Wake, REM and NREM amounts. Since only three animals contributed to these plots, we represent individual animals with thin grey lines instead of using error bars. Due to the small sample size, we did not conduct a statistical analysis. However, we agree with the reviewer that this presentation more effectively illustrates the transient increase in REM2 across all animals following NOE.

-The behavioral schematics from figure s1 should be moved to figure 1 as this adds valuable context.

Rev #1.2. We have modified Figure 1 moving panels A, B, and D from Supplementary 1 to Figure 1, and changes in the main text were made accordingly. To avoid including too many panels in Fig.1, we moved old Fig.1 C, D and F into Fig. S1, now titled: Assessment of auditory function in the ferret model: baseline assessments and long-term performance in operant gap detection.

-In general, the figures should be edited for visibility. In places the font sizes are different across panels, e.g. figures 3 and 4. In Figure 3B, middle, there are some graphics that appear to be a screenshot artifact that should be removed.

Rev #1.3. Figures have been further edited, artefacts (for example in Figure 3B) have been removed, and font sizes across panels and axes have been checked and updated as necessary. Specifically: axis labels in Fig. 3A, Fig. 4G and in the insets in Fig. 3C have been altered, and minor changes have been made in supplementary Figures S2-6, 10 and 15.

-The components that make up the tinnitus index calculation, and the assumptions of the TI, should be described verbally in the results section, as this gives important context to the data shown in figures.

Rev #1.4. As requested, the components of the tinnitus index calculation and the assumptions of the TI calculation have been described and summarised in the results section.

Line 556: “The tinnitus index was calculated as the sum of three metrics obtained by comparing the values before and after the noise overexposure, with positive values indicating changes in line with tinnitus development (see Methods).

$$\mathrm{Eq}\left( 1 \right): TI=M\left( cont \right)+M\left( thresh \right)+ M\left( silence \right)$$

The metrics for operant gap detection performance refer to the change in the ability of an animal to detect the continuous sound (no gap) across all tested stimulus frequencies and to the change in gap-detection threshold across all stimuli (Eq(2) and Eq(3) in the Methods). The metric for operant silence detection refers to the change in silence detection ability (percent correct score in silence trials) after noise overexposure (Eq(4) in Methods).”

-In the discussion, lines 897-898 contain a typo.

Rev #1.5. Deleted “The first scientific evidence”

**Reviewer #3:** The authors present behavioral and electrophysiological data investigating the link between tinnitus and sleep in 7 adult female ferrets, with 3 animals receiving long term EEG recordings and from 2 (+1) animals, auditory cortex activity was recorded with sharp electrodes. This resulted in three single cases, that could be described over six months in great detail. The manuscript is already revised and most concerns regarding the methodology and results have been accounted for.
Overall I suggest a major revision, especially in the Introduction and Discussion sections, in the latter many references (after 68) are missing.

Major points:
One major point that I am missing from the manuscript is the introduction to the different neurophysiological models of tinnitus development and the introduction to sleep and sleep related measurements (polysomnography). Both are relevant for the understanding of the used methods and the interpretation of the results.

Rev #3.1. The introduction section has been edited by adding a section on the main neurophysiological models of tinnitus development and an introduction to sleep and polysomnography.

Line 59: “Three main models have been implicated in the development of tinnitus, which attempt to explain the changes that occur in the brain following a cochlear insult: altered lateral inhibition, homeostatic plasticity, and stochastic resonance (reviewed in [19]). In the lateral inhibition model, tinnitus is caused by disinhibition of frequency channels adjacent to deafferented channels (edge effect; [20]); in the homeostatic plasticity model by an increase in spontaneous activity due to an increase in central neuronal gain [21], and, in the stochastic resonance model, by increased noise in a recurrent neural network, leading to subthreshold auditory signals becoming detectable [19].

Tinnitus is associated with functional changes in widely distributed brain regions, including both auditory and non–auditory areas [22–28], many of which exhibit a dramatic modulation in their spatiotemporal activity across vigilance states: for example, wakefulness is dominated by high-frequency, low-amplitude oscillatory brain activity, whereas sleep is dominated by slower, high-amplitude oscillations [29–31].”

Line 93: “We tracked behavioural markers of noise–induced tinnitus and hearing impairment in a ferret model at one week and six months after noise overexposure and, in parallel, assessed the sleep–wake pattern, as well as spontaneous and auditory–evoked EEG activity across vigilance states. In a similar manner to polysomnography, which is based on electromyogram (EMG) recordings and EEG recordings of global brain activity to assess human sleep and sleep disorders [4,29,40], we used EEG and EMG recordings for vigilance state scoring as previously established in rodents (e.g. [14,41]) and ferrets [42].”

For the reader, it would improve the readability, if instead of post1 and post2 the real timepoints are presented in the text and Figures. In some of the supplements, this is already the case and improves the intelligibility significantly.

Rev #3.2. This is a good idea. We now refer in the text and figures to the Post1 and Post2 timepoints as ‘1 week’ and ‘6 months’.

L888ff: Add here also the development aspect from the introduction. This is relevant, as you have early and late recording timepoints. Also compare your behavioral model of tinnitus identification to other behavioral animal models, including conditioning and reflexive models.

Rev #3.3. The Discussion has been edited to include the different models of tinnitus development and a comparison of our behavioural methods of tinnitus identification (operant gap and silence detection) with other reflexive models.

Line 1004: “Cochlear damage after noise overexposure can lead to a compensatory increase in neuronal excitability that restores evoked activity. This can occur through mechanisms such as homeostatic plasticity [21] or adaptive stochastic resonance [87], processes that can themselves drive tinnitus development [19,21,87]. Elevated cortical activity has been reported in human tinnitus sufferers [88,89] and in a range of animal models of induced tinnitus (chinchillas: [86], cats: [90], guinea pigs: [91,92], gerbils [93,94], rats: [95], and mice: [96]), including our own preliminary results (Fig S15).

The increased evoked activity we observed in the first assessment after NOE could be explained by adaptive stochastic resonance, which operates on short timescales [19]. In contrast, sustained tinnitus six months after NOE, especially in the context of higher levels of hearing loss, is more likely based on plastic changes [97] relating to homeostatic plasticity and alterations in central gain [21].

Assessments of tinnitus in our study are based on positive operant behavioural tasks rather than reflexive behaviours like the acoustic startle reflex, which show habituation that is particularly rapid in carnivores and primates, including humans [98]. However, there are further important differences in tinnitus identification based on operant and reflexive paradigms: for example, reflexive paradigms, such as classical assessment of the gap-induced inhibition of the behavioural startle response (GPIAS, [99,100]) make it challenging to separate tinnitus from hearing loss, whereas operant paradigms can be more sensitive to differences between these conditions (e.g. [55]). On the other hand, operant paradigms have a learning component that may affect the results (reviewed in [101]). For this reason, we completed extensive baseline training in all operant paradigms before testing animals for tinnitus (see methods).

Please check for the missing references after 68. Please also increase your comparisons of ABR shifts to more than one reference (69) for animals and humans (70).
For all references after 68, I cannot decide if they are appropriate or missing points. Please double-check.

Rev #3.4. We deeply apologize for the oversight on our part in submitting the previous revised version of the manuscript with the last third of the references missing. We can confirm that the reference list is complete.

To avoid including a long list of citations, we now use two reviews (Shore et al., 2016 and Baguley, 2002) that describe the variable effects of noise trauma on tinnitus and hearing impairment in experimental animals and the tinnitus heterogeneity in humans, respectively (Lines 964-967).

Lines 964-967: “Interestingly, we show that the measures of the degree of tinnitus or its perception by the animal, as well as hearing impairment following noise overexposure are highly idiosyncratic, in line with variable effects of noise trauma seen in other animal models and the tinnitus heterogeneity that is characteristically found in humans (for example reviewed in [76] and [77], respectively).”

L 955: An important tinnitus animal model is the Mongolian gerbil, please add references here.

Rev #3.5. References added to Line 1009 [Tziridis et al., 2015; Jeschke et al., 2021] and to the reference list.

Tziridis K, Ahlf S, Jeschke M, Happel MF, Ohl FW, Schulze H. [Noise Trauma Induced Neural Plasticity Throughout the Auditory System of Mongolian Gerbils: Differences between Tinnitus Developing and Non-Developing Animals.](https://pubmed.ncbi.nlm.nih.gov/25713557/) Front Neurol. 2015 Feb 10;6:22. doi: 10.3389/fneur.2015.00022.

Jeschke M, Happel MFK, Tziridis K, Krauss P, Schilling A, Schulze H, Ohl FW [Acute and Long-Term Circuit-Level Effects in the Auditory Cortex After Sound Trauma.](https://pubmed.ncbi.nlm.nih.gov/33469416/) Front Neurosci. 2021 Jan 5;14:598406. doi: 10.3389/fnins.2020.598406.

L964ff: Here you could speculate about the circadian influence on hearing impairment. In rodents, it has been shown to be most relevant for hearing loss and tinnitus development: DOI 10.3389/fnins.2022.830703; 10.1121/1.5132290

Rev #3.6. The reviewer raises an interesting point for discussion about the circadian modulation of tinnitus and hearing loss. Nocturnal animals, such as mice, are more sensitive to noise overexposure when the stimulus is applied during the night (Meltser et al., 2014), whereas the converse happens in diurnal animals like gerbils (Grimm et al., 2022), indicating a higher sensitivity to auditory insult during the active phase. We have addressed this in the last paragraph on page 41 (lines 1037-1052) by incorporating circadian modulation in the discussion and the studies by Meltser et al., (2014) and Grimm et al. (2022) have been added to the reference list.

Line 1037-1053: “The main limitations of this cross-case study are the small number of animals with chronic implants, and the absence of specific *ad hoc* controls. In addition, the elevation of ABR thresholds six months after NOE could have been a confounding factor as the relationship between hearing loss and tinnitus has long been recognised (e.g. [104], reviewed in [105]), even when only a hidden hearing loss is present [106,107]. The time of day also affects the impact of noise trauma on tinnitus and hearing loss. Nocturnal animals, such as mice, are more sensitive to noise overexposure when the stimulus is applied during the night [108], whereas the converse happens in diurnal animals like gerbils [109], indicating a stronger effect of the auditory insult during the active phase. Interestingly, tinnitus severity in gerbils is highest when the sound overexposure is conducted at the most sensitive time (5 pm), which is also when the largest noise-induced hearing loss is seen [109]. Ferrets are crepuscular animals, more active at dawn and dusk, and noise overexposure in our experiments was always conducted during the light period. Future studies varying the parameters used for noise overexposure, including the time of day when noise overexposure is applied, will decipher whether it is possible to disentangle hearing loss and tinnitus in the ferret model and its respective interaction with sleep states and circadian rhythms.”

Grimm J, Schulze H, Tziridis K (2022) Circadian Sensitivity of Noise Trauma-Induced Hearing Loss and Tinnitus in Mongolian Gerbils. Front. Neurosci. 16, <https://doi.org/10.3389/fnins.2022.830703>

Meltser, I., Cederroth, C. R., Basinou, V., Savelyev, S., Lundkvist, G. S., and Canlon, B. (2014). TrkB-mediated protection against circadian sensitivity to noise trauma in the murine cochlea. Curr. Biol. 24, 658–663. doi: 10.1016/j.cub.2014.01.047.

L967: reference style changed here. For hidden hearing loss, also DOI 10.1111/ejn.15334 is important.

Rev #3.7. Thank you for highlighting this paper, which is pertinent to the potential link between hidden hearing loss and tinnitus. Tziridis et al., (2021) has been added.

Tziridis K, Forster1 J, Buchheidt-Dörfler I, Krauss P, Schilling A, Wendler O, Sterna E, Schulze H (2021) Tinnitus development is associated with synaptopathy of inner hair cells in Mongolian gerbils. Eur J Neurosci. 2021;54:4768–4780.

Minor points:
L XXX: remove “we”

Rev. # 3.8. “we the” has been removed from Line 194

L XXX: “no gap” should be “no-gap”

Rev #3.9. We have changed “no gap” by “no-gap” in the instances where it is used as an adjective.

L 656: Fig. 6A-C should be Fig. 2A-C

Rev #3.10. We thank the reviewer for spotting this. We have changed Fig 6A-C to Fig 2A-C in the revised 2 version (Line 706).

**Reviewer #4:** This is an interesting paper and would be of interest to many tinnitus researchers. However the data are over-interpreted. The authors have made changes to respond to reviewers but need to make additional changes in order for the paper to be acceptable for publication.
My biggest problem with the data from the 3 animals is that there is no consistent pattern (see also fig S4) at any of the sleep states and the study lacks sufficient number of animals to say anything meaningful. It does show that these studies are possible and it warrants further investigation but authors should temper conclusions throughout the manuscript.

Rev. # 4.1. We thank the reviewer for their interest in the paper and for their suggestions. We have tempered our conclusions throughout the paper, by making the number of animals used clear in each part of this study, and by stressing that this work is a cross-case study, based on individual animals. Specifically, we have added a new paragraph to the Methods (Line 123) to clarify the number of animals used in each part of the study, including one ferret that was only used in the baseline behaviour and one naïve animal used as a control for the terminal cortical recordings. In addition, we have added the animal sample sizes to the figure legends (See also responses to Rev. #4.2, #4.3, #4/9, and #4.10).

Line 123: “A total of nine ferrets were used in this study. Eight animals were used for tinnitus assessment in the baseline condition and then exposed to noise. One animal was subsequently excluded from the experiment due to health issues unrelated to the procedure and no behavioural data obtained in this animal after NOE were included in the analysis. A subset of four animals were implanted for chronic recordings but only three were used for long-term assessment. Two animals were assessed in all paradigms, whereas one was only used for ABR and operant gap detection measurements (since the experiment had to be terminated prematurely due to health reasons unrelated to the experimental protocol). The animals were chronically implanted with cortical EEG and EMG electrodes to assess sleep–wake architecture and spatiotemporal patterns of brain activity before and after noise overexposure. The longitudinal design of this study spanning more than 9 months allowed each animal to act as its own control by comparing the results before and after noise overexposure. We did not use specific controls comprising implanted animals without noise overexposure, since no changes in behaviour or neural activity were found in previous ferret experiments following similar surgical procedures and/or intracranial implantation [46,47]. One additional naïve animal was used as a control to compare physiological correlates of tinnitus with two out of the three ferrets implanted for chronic recordings.”

Abstract and throughout: As the 3 animals that were investigated for sleep patterns all had tinnitus the statement that “Animals that developed signs of tinnitus consistently developed sleep impairments, suggesting a link between the emergence of noise–induced tinnitus and sleep disruption” is an overstatement. It may be the hearing loss that causes the problem not tinnitus.
It should also be made clear in the abstract that only 3 animals were investigated.

Rev. # 4.2. As the Reviewer recommends, we have mentioned the number of animals used in the study in the abstract and throughout the paper and modified the relevant sentence in the abstract with regards to the possibility of hearing loss in these animals.

Lines 20-39: “

Subjective tinnitus is a phantom auditory perception in the absence of an actual acoustic stimulus that affects 15% of the global population. In humans, tinnitus is often associated with disturbed sleep and, interestingly, there is an overlap between the brain areas involved in tinnitus and regulation of NREM sleep. We used eight adult ferrets exposed to mild noise trauma as an animal model of tinnitus. We assessed the phantom percept using two operant paradigms sensitive to tinnitus, silent gap detection and silence detection, before and, in a subset of animals, up to six months after the mild acoustic trauma. The integrity of the auditory brainstem was assessed over the same period using auditory brainstem response recordings.

Following noise overexposure, ferrets developed lasting, frequency–specific impairments in operant behaviour and evoked brainstem activity. To explore the interaction between sleep and tinnitus, in addition to tracking the behavioural markers of noise–induced tinnitus and hearing impairment after noise overexposure, we evaluated sleep–wake architecture and spontaneous and auditory–evoked EEG activity across vigilance states. Behavioural performance and auditory–evoked activity measurements after noise overexposure suggested distinct degrees of tinnitus and hearing impairment between individuals. Animals that developed signs of tinnitus consistently developed sleep impairments, suggesting a link between the emergence of noise–induced hearing loss and/or tinnitus and sleep disruption. However, neural markers of tinnitus were reduced during sleep, suggesting that sleep may transiently mitigate tinnitus.”

Introduction:
It is mentioned that “We tracked behavioural markers of noise–induced tinnitus and hearing impairment in a ferret model over a period of six months after noise overexposure” This suggests regular testing whereas testing was only done at two timepoints. This should be made more clear. In addition only 3 of the animals were tested at the 6 months timepoint. This is mentioned in results but not made clear in introduction or methods.

Rev. # 4.3. As requested, we have changed the sentence to clarify that the testing was done at two timepoints, one week and six months after noise overexposure. We also added in the introduction that only 3 of the animals were tested at six months.

Lines 93-103: “We tracked behavioural markers of noise–induced tinnitus and hearing impairment in a ferret model at one week and six months after noise overexposure and, in parallel, assessed the sleep–wake pattern, as well as spontaneous and auditory–evoked EEG activity across vigilance states. In a similar manner to polysomnography, which is based on electromyogram (EMG) recordings and EEG recordings of global brain activity to assess human sleep and sleep disorders [4,29,40], we used EEG and EMG recordings for vigilance state scoring as previously established in rodents (e.g. [14,41]) and ferrets [42].

From the initial group of eight ferrets exposed to noise and tested in behavioural paradigms, three ferrets were implanted with recording electrodes and tested again six months after noise overexposure. Behavioural performance …”

Same as Abstract comments: “Animals developing tinnitus also exhibited sleep impairments, suggesting a link between the emergence of noise–induced tinnitus and sleep disruption.” This is misleading as no animals without tinnitus were investigated for sleep patterns.

Rev. # 4.4. Sentence has been changed accordingly.

Lines 105-107. “While all implanted animals showed signs of hearing loss, those developing the highest levels of tinnitus severity also exhibited sleep impairments, suggesting that the emergence of noise–induced hearing loss and tinnitus is associated with sleep disruption.”

Methods:
New figure 6 was not available and also no figure 5? Although there is no reference to a figure 5?

Rev. # 4.5. There is no Figure 5 or 6 in the previous submitted manuscript. As Reviewer 3 pointed out (see Rev.#3.10), we incorrectly referred to Fig. 6A-C instead of Fig. 2A-C. This has been corrected in the present version (Line 706).

The additional data and figure showing spontaneous firing rate increase in the tinnitus animals versus naïve control is useful but I suggest the authors reference “Meredith MA, Keniston LP, Allman BL. Multisensory dysfunction accompanies crossmodal plasticity following adult hearing impairment. Neuroscience. 2012 Jul 12;214:136-48” who showed this as well following hearing loss and therefore it cannot be stated this is a marker for tinnitus.

Rev. # 4.6. We thank the reviewer for highlighting this paper; it has been now cited in the discussion.

Line 1027.

“It is possible that animals displaying signs of tinnitus in our study also developed other noise–induced conditions, such as elevated sensitivity to environmental sounds or hyperacusis. Our animals also developed different degrees of hearing loss and an increase in both spontaneous and driven activity in auditory cortical neurons, as has been reported in ferrets presenting moderate hearing loss following injections of the ototoxic drug kanamycin [102]. Although hearing loss and hyperacusis would not account …”

Meredith MA, Keniston LP, Allman BL. Meredith Multisensory dysfunction accompanies crossmodal plasticity following adult hearing impairment. Neuroscience. 2012 Jul 12;214:136-48. doi: 10.1016/j.neuroscience.2012.04.001.

Line 194: remove “we the” from “models, we the noise was applied”

Rev. # 4.7. “we the” has been removed from Line 218.

Line 539: “an effect visible throughout the experiment (Figs 1A)” Figure 1A doesn’t show an effect just experimental design?

Rev. # 4.8. The reviewer is absolutely right. It should say Fig S1A. This has been updated in the present version (Line 574).

Line 642: “Although ABRs were not performed in sham-operated control cases”. I thought no sham controls were performed? All 7 animals were exposed to AT?

Rev. # 4.9. We apologise for the confusing phrasing. This sentence referred to the cited study, where sham-operated controls were used. In our study, all implanted animals underwent the noise overexposure procedure and no sham-operated controls were included. We revised the manuscript as follows:

Line 690: “The surgical procedure undergone by the three ferrets is unlikely to have contributed to the observed hearing impairment since previous experiments [46] indicate no effects of comparable cranial surgery on auditory cortical responses in adult ferrets of the same age as in the current study when tested 30 months later.”

Line 694: “also showed reduced sleep disruption (3B)” Figure 3 B shows gap detection.
Legends for all figures should show n for each group. Please explain the colour coding. I also assume the symbols represent different animals. However there is an upside down triangle in fig 1B baseline that is not visible in 1 week?

Rev. # 4.10. We corrected the figure reference. We also included a description of the colour coding in all legends if not already explained in the figure panels (blue for baseline, red for 1 week and orange for 6 months following noise overexposure). Animal markers are now described as such in all relevant figure legends. We previously stated that seven animals were used for behavioural and ABR assessments. However, for baseline assessment of behaviour (operant gap detection and operant silence detection) eight animals were initially used, but one animal was subsequently excluded from the experiment due to health issues unrelated to the procedure and no behavioural data obtained in this animal after NOE were included in the analysis. We now explain this in the methods and have added animal numbers to all figure legends.

Line 1149: “ Symbols represent individual animals (n=8 baseline, n=7 one week, n=3 six months).”

Line 1163: “(A) Experimental timeline for implanted animals. Three ferrets were assessed …”

Line 1255: “Fig S1. Assessment of auditory function in ferrets: baseline assessments and long-term performance in operant gap detection. (A) Baseline performance in the operant gap detection task showing the proportion of left (-30°) responses by gap length (n=7).”

Regarding Figure 1 E: what does the asterisk represent? Are the 3 groups different from each other under NBN condition? They seem identical and the difference not noteworthy

Rev. # 4.11. Asterisk in panel E represents statistical significance at p<0.05 between proportion of correct responses at baseline and at six months following noise overexposure. This information has been added to the figure legend.

Line 1154. “ …sessions. Asterisk represents statistical significance at p<0.05 between proportion of correct responses at baseline and at six months following noise overexposure.”

It would be useful to show tinnitus index for all animals where available. Not just for the 3 animals that had sleep analysed. It seems all animals developed tinnitus? This is unexpected as other animals models show around 50% of animals to develop tinnitus, similar to humans where not everyone with a hearing loss develops tinnitus.

Rev. # 4.12. We conducted long-term behavioural and auditory brainstem response (ABR) assessments for the three animals that also provided chronic sleep data, as detailed in the manuscript. For the remaining four animals, no sleep recordings were obtained, and assessments were only performed before and one week after noise overexposure (NOE). Due to the limited timeframe for data collection, it was not possible to determine whether lasting tinnitus had developed in these animals; therefore, a tinnitus index was not generated. Nonetheless, these animals exhibited considerable variability in behavioural markers of tinnitus, such as gap detection thresholds (old Fig. 1B that corresponds to Fig. 1E in the revised 2 version of the manuscript) and silence detection ability (old Fig. 1E, which is now Fig. 1F) one week after NOE. Based on this, we conclude that NOE successfully induced signs of tinnitus in some, but not all, animals.

Figure 4 E and F do not show the 6 month data for case 2 and 3?

Rev. # 4.13. Thank you for pointing this out. We state in the discussion: ‘Six months after NOE, Ferret 2 showed qualitative evidence for further increased auditory-evoked activity in both frontal and occipital derivations (Fig S12). Due to decreased signal quality in the Six months assessment, this could not be quantitatively verified.’ We have now added the following to the figure legend of Fig.4:

Line 1230: “(**F**) Depiction as in **E** but for occipital EEG configuration. Asterisks for panels E-F represent statistical significance p values *<0.05, **<0.01, ***<0.001 (GLMM). Each panel depicts one ferret. Panels left to right: Ferret 1, Ferret 2, Ferret 3). Due to decreased signal quality in the six months post NOE assessment in Ferrets 2 and 3, AERs could not be quantitatively assessed for these timepoints.”

Discussion:
“we provided the first empirical evidence provided initial evidence supporting the idea that tinnitus emergence coincides with emergence of sleep disruption.” This is an overstatement. Tinnitus development was only monitored at two timepoints, long time apart.

Rev. # 4.14. The text has been changed following the reviewer’s comment to more precisely reflect the results of our cross-case study.

Lines 949-953: “By combining this with the assessment of sleep architecture and spatiotemporal brain activity, our measurements at two time points, one week and six months, following noise overexposure provide initial evidence that tinnitus emergence might coincide with emergence of sleep disruption.”

With regards to tinnitus studies in humans it would be worthwhile to discuss whether these studies investigated people bothered by their tinnitus as their stress and anxiety levels are likely to affect sleep patterns.
“More specifically, elevated cortical activity has been reported in humans [81,82] and in a range of animal models of induced tinnitus (chinchillas: [80], cats: [83], guinea pigs: [84,85], rats: [86], and mice: [87]), including our own preliminary results (Fig S16).” See my previous comment re Meredith et al. It is also associated with hearing loss per se.

Rev. # 4.15. One of the main problems of studying tinnitus and sleep disturbance in humans is the common association of both, as we stated in the introduction (see references 3-11), with inconclusive results concerning the relationship between tinnitus severity and sleep changes. The challenge of investigating this relationship in humans was one of the main motivations of using an animal model to study the association between tinnitus and sleep. Our experimental animals behaved similarly to normal ferrets, without any obvious signs of stress or anxiety, as we stated in our paper:

(Lines 687-694): “The seven animals presented specific behavioural and hearing impairments without any noticeable general change in demeanour or wellbeing following noise overexposure. No changes in body weight, bowel habits, fur aspect, or social interaction were observed that could indicate potential noise overexposure related distress (see Methods). The surgical procedure undergone by the three ferrets is unlikely to have contributed to the observed hearing impairment since previous experiments [46] indicate no effects of comparable cranial surgery on auditory cortical responses in adult ferrets of the same age as in the current study when tested 30 months later.”

We have added Meredith et al. (2012) in Lines 1027-1036 (as reported in comment Rev. #4.6)

“It is possible that animals displaying signs of tinnitus in our study also developed other noise–induced conditions, such as elevated sensitivity to environmental sounds or hyperacusis. Our animals also developed different degrees of hearing loss and an increase in both spontaneous and driven activity in auditory cortical neurons, as has been reported in ferrets presenting moderate hearing loss following injections of the ototoxic drug kanamycin [102] [Meredith et al., 2012]. Although hearing loss and hyperacusis would not account for all the behavioural deficits observed in this study, such as impaired silent gap detection, it cannot be ruled out that hyperacusis was an additional consequence of noise overexposure and contributed to sleep impairments. Indeed, hyperacusis has been suggested as common factor in both tinnitus and insomnia [103].”

The increased hearing loss at 6 months is very surprising. Other animal models do not show this following an AT. Hearing thresholds are generally stable after 1 or 2 weeks. Hence statement “The main limitations from this case study are the absence of temporary shifts in ABRs following noise overexposure” is incorrect as immediate loss was not measured.

Rev. # 4.16. Hearing loss was measured one week after noise overexposure, and again six months later. It was unexpected that the hearing loss increased at six months, suggesting that the parameters that we used for noise overexposure, which were similar to those used for rodents, may need to be re-evaluated in ferrets (Lines 959-968). The fact that measures of tinnitus and hearing loss varied between animals (with a predominance of one or the other in each animal) suggest that the impact of noise overexposure depends on individual variation that needs to be taken into account, in line with previous results in other animal models (for example reviewed in Shore et al., 2016) and the tinnitus heterogeneity observed in humans (Beukes et al., 2021).

The sentence “The main limitations …” in Line 1037 has been modified as follow:

The main limitations of this cross-case study are the small number of animals with chronic implants, and the absence of specific *ad hoc* controls. In addition, the elevation of ABR thresholds six months after NOE could have been a confounding factor as the relationship between hearing loss and tinnitus has long been recognised (e.g. [104], reviewed in [105]), even when only a hidden hearing loss is present [106,107]. The time of day also affects…”

Conclusion section needs to be tempered as “states provided evidence for a bi–directional interaction between tinnitus and natural brain state dynamics” is an overstatement of the results.

Rev. # 4.17. This sentence has been modified to include the word “suggest” instead of “provide” evidence.

Lines 1094-1097: “A combination of tinnitus and hearing assessments, vigilance state analysis, and measurement of spontaneous and auditory–evoked EEG activity across vigilance states in three animals suggest a bi–directional interaction between tinnitus and natural brain state dynamics.”

7. PLOS authors have the option to publish the peer review history of their article ([what does this mean?](https://c05y1x9s.r.us-east-2.awstrack.me/L0/https:%2F%2Fjournals.plos.org%2Fplosone%2Fs%2Feditorial-and-peer-review-process%23loc-peer-review-history/1/010f019203314c6f-c923db4e-3290-458c-baba-e29c59bbc5f6-000000/jDE1kCaorXkK44XeFCSAP8q7Wvc=177)). If published, this will include your full peer review and any attached files.

If you choose “no”, your identity will remain anonymous but your review may still be made public.

**Do you want your identity to be public for this peer review?** For information about this choice, including consent withdrawal, please see our [Privacy Policy](https://c05y1x9s.r.us-east-2.awstrack.me/L0/https:%2F%2Fwww.plos.org%2Fprivacy-policy/1/010f019203314c6f-c923db4e-3290-458c-baba-e29c59bbc5f6-000000/3OV4wCPofaPNJjlKE5wFGtaSoeg=177).

Reviewer #1: No

Reviewer #3: **Yes:** Konstantin Tziridis

Reviewer #4: No

[NOTE: If reviewer comments were submitted as an attachment file, they will be attached to this email and accessible via the submission site. Please log into your account, locate the manuscript record, and check for the action link "View Attachments". If this link does not appear, there are no attachment files.]

While revising your submission, please upload your figure files to the Preflight Analysis and Conversion Engine (PACE) digital diagnostic tool, [https://pacev2.apexcovantage.com/](https://c05y1x9s.r.us-east-2.awstrack.me/L0/https:%2F%2Fpacev2.apexcovantage.com%2F/1/010f019203314c6f-c923db4e-3290-458c-baba-e29c59bbc5f6-000000/2uLmIbO13TOeiYywKCOdjpmqC2c=177). PACE helps ensure that figures meet PLOS requirements. To use PACE, you must first register as a user. Registration is free. Then, login and navigate to the UPLOAD tab, where you will find detailed instructions on how to use the tool. If you encounter any issues or have any questions when using PACE, please email PLOS at [figures@plos.org](mailto:figures@plos.org). Please note that Supporting Information files do not need this step.
